# Targeting tumor exosomal circular RNA cSERPINE2 suppresses breast cancer progression by modulating MALT1-NF-𝜅B-IL-6 axis of tumor-associated macrophages

**DOI:** 10.1186/s13046-023-02620-5

**Published:** 2023-02-17

**Authors:** Boxuan Zhou, Zhaohong Mo, Guie Lai, Xiaohong Chen, Ruixi Li, Runxin Wu, Jia Zhu, Fang Zheng

**Affiliations:** 1 Department of Breast Surgery, the First Affiliated Hospital of Gannan Medical University, Ganzhou, 341000 China; 2 Medical Research Center and Guangdong Provincial Key Laboratory of Malignant Tumor Epigenetics and Gene Regulation, Sun Yat-Sen Memorial Hospital, Sun Yat-Sen University, Guangzhou, 510120 China; 3 Department of Hepatobiliary Surgery, the Third Affiliated Hospital, Sun Yat-Sen University, Guangzhou, 510630 China; 4 Department of Laboratory, the First Affiliated Hospital of Gannan Medical University, Ganzhou, 341000 China; 5 Department of Hepatobiliary and Pancreatic Surgery, The Eighth Affiliated Hospital, Sun Yat-Sen University, Shenzhen, 518033 China; 6 Zhongshan School of Medicine, Sun Yat-Sen University, Guangzhou, 510080 China; 7 Department of Breast Surgery, the First Affiliated Hospital of Nanchang University, Nanchang, 330000 China

**Keywords:** Breast cancer, cSERPINE2, Tumor-associated macrophages, Exosome, Nanoparticles

## Abstract

**Background:**

Circular RNAs (circRNAs) have important regulatory functions in cancer, but the role of circRNAs in the tumor microenvironment (TME) remains unclear. Moreover, we also explore the effects of si-circRNAs loaded in nanoparticles as therapeutic agent for anti-tumor in vivo.

**Methods:**

We conducted bioinformatics analysis, qRT-PCR, EdU assays, Transwell assays, co-culture system and multiple orthotopic xenograft models to investigate the expression and function of circRNAs. Additionally, PLGA-based nanoparticles loaded with si-circRNAs were used to evaluate the potential of nanotherapeutic strategy in anti-tumor response.

**Results:**

We identified oncogene SERPINE2 derived circRNA, named as cSERPINE2, which was notably elevated in breast cancer and was closely related to poor clinical outcome. Functionally, tumor exosomal cSERPINE2 was shuttled to tumor associated macrophages (TAMs) and enhanced the secretion of Interleukin-6 (IL-6), leading to increased proliferation and invasion of breast cancer cells. Furthermore, IL-6 in turn increased the EIF4A3 and CCL2 levels within tumor cells in a positive feedback mechanism, further enhancing tumor cSERPINE2 biogenesis and promoting the recruitment of TAMs. More importantly, we developed a PLGA-based nanoparticle loaded with si-cSERPINE2, which effectively attenuated breast cancer progression in vivo.

**Conclusions:**

Our study illustrates a novel mechanism that tumor exosomal cSERPINE2 mediates a positive feedback loop between tumor cells and TAMs to promote cancer progression, which may serve as a promising nanotherapeutic strategy for the treatment of breast cancer.

**Supplementary Information:**

The online version contains supplementary material available at 10.1186/s13046-023-02620-5.

## Background

Globally, breast cancer affects more women than any other type of cancer and is the leading cause of cancer-related death for women [1, 2]. Although active surgery combined with radiotherapy, chemotherapy and hormone therapy have achieved benefits, the treatment efficacy is still limited for some breast cancer patients [3]. The advent of targeted therapy has transformed the concept of cancer treatments, where both early and advanced breast cancer can be targeted by specific proteins or genes to improve clinical outcomes [4]. Hence, more novel targets are urgently needed to be discovered to optimize current therapies and address acquired resistance.

The tumor microenvironment (TME) is an essential precondition for the occurrence and progression of tumor [5–7]. As one of the most abundant stromal cell types in the TME, tumor-associated macrophages (TAMs) function in response to various microenvironmental signals [8]. For example, colorectal cancer cell-conditioned macrophages are characterized by a mixed M1/M2 phenotype and regulate the EMT program to enhance tumor cell migration and invasion [9]. However, the crosstalk between TAMs and cancer cells is extremely sophisticated. Accordingly, exploring pivotal mechanisms underlying the communication between TAMs and cancer cells may aid in the development of effective treatment strategies to improve the outcome of breast cancer patients.

Extracellular vesicles (EVs) are composed of lipid bilayer membrane-enclosed particles released from most cell types. They are classified into small EVs (< 200 nm in diameter), medium/large EVs(> 200 nm) based on the different sizes and the mode of biogenesis pathways, termed exosomes, microvesicles, microparticles, ectosomes, oncosomes, apoptotic bodies according to the International Society for Extracellular Vesicles (ISEV) [10]. But exosomes which are originated from the endosomal compartment, are membrane-enclosed vesicls 30 nm-150 nm in diameter referred to as small EVs [11]. Exosomes contain abundant bioactive molecules, including nucleotides, metabolites and lipids from their parental cells [10]. Increasing evidences have established that exosomes function as intercellular mediators by transferring functional cargoes to, or engage membrane receptor-mediated signaling in recipient cells [12]. Moreover, recent studies suggest that circRNAs can be encapsulated within exosomes to regulate tumor growth, angiogenesis and metastasis, indicating that TME may be affected by exosomal circRNAs [13, 14].

Serpin family E member 2 (SERPINE2), belonging to the serine protease inhibitor superfamily, usually functions as a secreted protein with anti-serine protease activity against thrombin, urokinase and plasminogen [15]. Moreover, SERPINE2 is involved in the progression of multiple cancers [15–20]. For instance, SERPINE2 overexpression promoted the metastasis of breast cancer by remodeling the tumor matrix [19]. Nevertheless, the current explorations of SERPINE2 cannot fully illustrate the diversity of its involvement in tumor development. Therefore, elucidation of the function of circRNAs arising from SERPINE2 is urgently needed.

Herein, we shown that SERPINE2-derived hsa_circ_0001103 (cSERPINE2) was notably elevated in breast cancer and was associated with poor clinical outcome. Interestingly, we found that cSERPINE2 reshaped the breast cancer immune microenvironment in vivo but had no effect on biological functions of tumor cells in vitro. Mechanistically, tumor exosomal cSERPINE2 was shuttled to TAMs and notably elevated MALT1 levels, enhancing the secretion of Interleukin-6 (IL-6) by activating the NF-$\upkappa$B pathway and leading to increased proliferation and invasion of breast cancer cells. More importantly, IL-6 in turn increased the EIF4A3 and CCL2 levels within tumor cells in a positive feedback mechanism, further enhancing tumor cSERPINE2 biogenesis and promoting the recruitment of TAMs. Moreover, the PLGA-based si-cSERPINE2 nanoparticles effectively attenuated breast cancer progression in vivo. Taken together, our study illustrates that tumor exosomal cSERPINE2 mediates a positive feedback loop between tumor cells and TAMs to promote cancer progression, which may serve as a promising nanotherapeutic strategy for the treatment of breast cancer.

## Methods

### Patient and tissue samples

Breast cancer tissues were obtained from surgical specimen archives of 136 patients from the First Affiliated Hospital of Gannan Medical University, between 2009 and 2012. The inclusion criteria of patients were as follow: i) Female; ii) Diagnosed as breast cancer by pathological confirmation; iii) No adjuvant treatment including chemotherapy, radiotherapy, hormone therapy or immunotherapy before surgery; iv) Complete clinical and pathological data. The patients had the exclusion criteria as follows: i) Male; ii) Combined with other tumors and serious diseases; iii) incomplete clinical and pathological data. Frozen tissues were used for quantitative reverse transcription PCR (qRT-PCR) analyses, while paraffin-embedded tissues were used for FISH and IHC analyses.

All samples were collected from patients with informed consent, and all related procedures were performed with the approval of the internal review and ethics boards of the First Affiliated Hospital of Gannan Medical University. The study was compliant with all relevant ethical regulations regarding research involving human participants.

### Exosome isolation, quantification and characterization

When equal cell numbers of tumor cells (MCF-7 and EO771 cells) were seeded and reached 70% confluency in 150 mm culture dishes, cells were washed with PBS and cultured in exosome-depleted media to isolate exosomes after 48–72 h of further culture as previously described [14, 21]. Briefly, equal volume of conditioned media were collected and centrifuged at 3000 rpm for 10 min, followed by a centrifugation step of 10,000 × g for 30 min at 4℃ to remove cellular debris. Subsequently, the supernatant was filtered through a 0.22- μm filter. Exosomes were ultracentrifuged at 100,000 × g for 90 min and resolved in 100 μl PBS.

For exosomes protein quantification, we used 10 × RIPA buffer to lyse exsomes. Then, micro BCA Protein Assay Reagent Kit (Cat#23,235, Invitrogen, USA) were used to quantificate the protein concentration of lysed exosomes. Additionally, exosomes were examined by electron microscopy using negative staining, and quantified using a NanoSight NS300 instrument (Malvern Instruments) equipped with NTA 3.0 analytical software.

### Animal studies and in vivo imaging

The animal study procedures were approved by the Animal Care and Use Committee of Sun Yat-sen University. 4–6 weeks-old female C57BL/6, *Csf1*^*op/op*^ and corresponding wide-type mice were randomized to each experimental group. For orthotopic transplantations, 10^6^ EO771 cells transduced with lentivirus carrying control or cSERPINE2 shRNA and with or without cSERPINE2 overexpression were resuspended in 100μl of PBS and injected into the fourth mammary fat pads on one flank of the mice.

In some experiments, intraperitoneal injection was performed with 200μg of IL-6 antibody or control IgG antibody three times per week as previously described [22]. For the exosome injection mouse breast cancer model, exosomes obtained from cells with a dose of approximately 10^10^ were resuspended in 100μl PBS per mouse, which was injected into mice every 3 days as previously described [23, 24].

The tumor volumes were calculated by the formula: volume = width^2^ × length/2. The maximum permitted tumor diameter of 20 mm in any dimension was never exceeded. We recorded the tumor volumes every 3 days. Additionally, mouse tumor burden was monitored by weekly bioluminescence imaging using the IVIS-200 Lumina Imaging System (Xenogen). Lung metastatic lesions were confirmed by histological analysis.

Additional methods information can be found in Supplementary Materials.

## Results

### Identification analysis of a novel circular RNA generated from the SERPINE2 gene

**Fig. 1:**
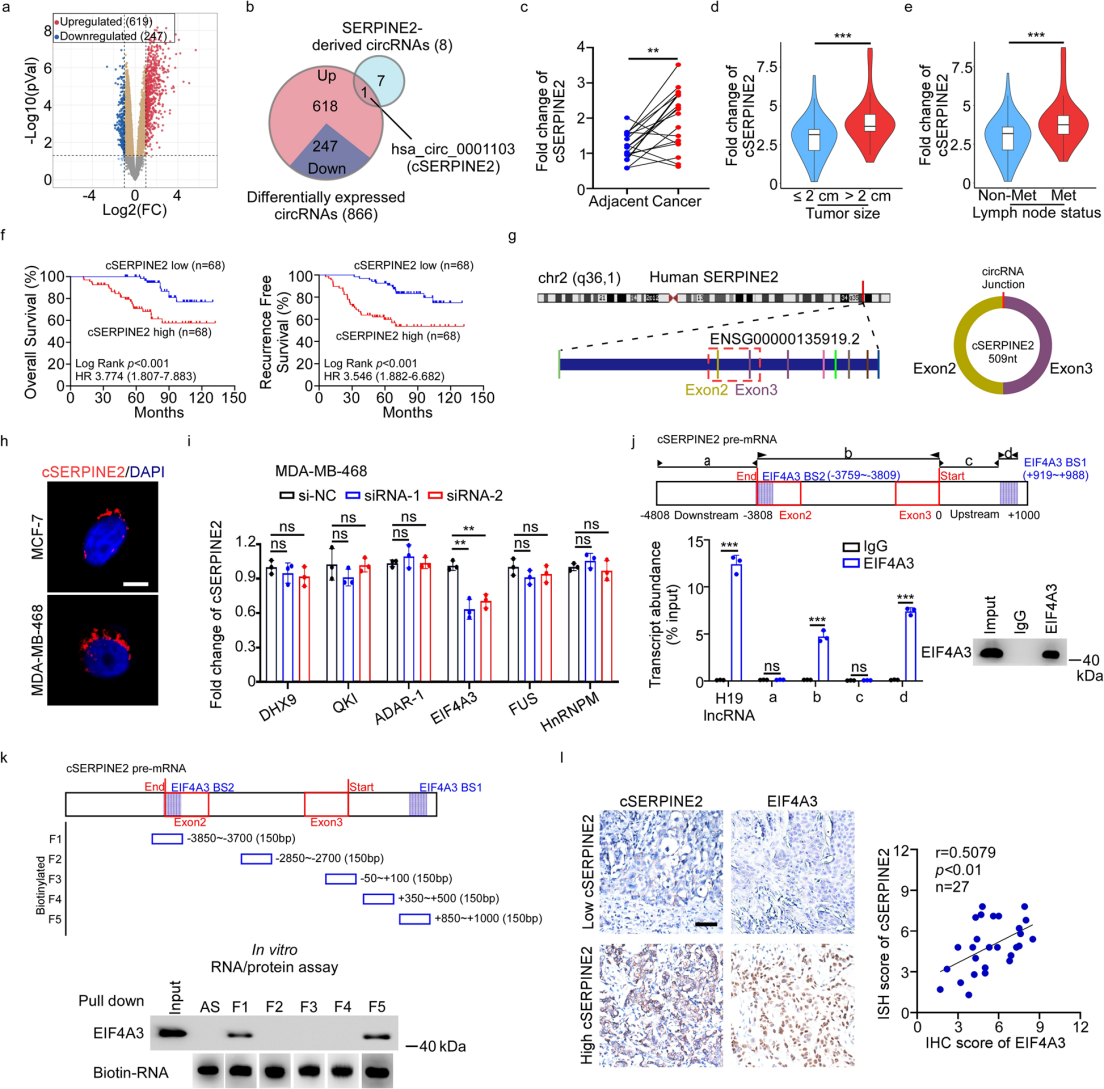
Analysis of SERPINE2-derived circRNAs in breast cancer. **a** Volcano plot showing the differentially expressed circRNAs in breast cancer tissues compared to matched adjacent tissues in GSE182471 dataset. **b** Venn diagram showing the intersection of differentially expressed circRNAs in GSE182471 dataset and SERPINE2-derived circRNAs. **c** The relative expression of cSERPINE2 in breast cancer tissues and matched adjacent tissues (*n* = 18). **d, e** 136 breast cancer patients were divided into different groups according to tumor size or lymph node metastasis status. The diagram showing cSERPINE2 expression in each group. **f** Kaplan–Meier analysis of the OS and RFS of breast cancer patients according to cSERPINE2 expression. **g** Diagram illustrating back-splicing of cSERPINE2 from the host gene SERPINE2.** h** Representative images of FISH analysis of cSERPINE2 in MCF-7 and MDA-MB-468 cells. Scale bar, 5 μm. **i** The relative expression of cSERPINE2 in MDA-MB-468 cells after knocking down DHX9, QKI, ADAR-1, EIF4A3, FUS or HnRNPM. **j** Putative binding sites of EIF4A3 in cSERPINE2 pre-mRNA predicted by CircInteractome database (upper), and RIP assay (lower) of interaction of EIF4A3 with cSERPINE2 pre-mRNA in MDA-MB-468 cells. The H19 lncRNA was used as the positive control. **k** A schematic diagram of five fragments of cSERPINE2 pre-mRNA (upper), and RNA pull-down analysis (lower) of the interaction between EIF4A3 and the above five fragments of cSERPINE2 pre-mRNA. **l** Representative images (left) of ISH analysis of cSERPINE2 expression and IHC analysis of EIF4A3 expression in breast cancer tissues, and a correlation analysis (right) between the cSERPINE2 and EIF4A3 expression. Scale bar, 50 μm. Data are presented as the means ± SD of three independent experiments. *P < 0.05, **P < 0.01, ***P < 0.001

A number of host genes of circRNAs play oncogenic roles in tumorigenesis, but it remains unclear whether oncogenes-derived circRNAs affect cancer progression. Given that SERPINE2 overexpression programmed breast cancer cells for vascular mimicry and contributed to distant metastasis [20], we wondered whether SERPINE2-derived circRNAs exerted influence on breast cancer progression. We first analyzed the expression of 8 SERPINE2-derived circRNAs in breast cancer and found that only hsa_circ_0001103 (cSERPINE2) was substantially upregulated (Fig. 1a, b). Consistently, the expression of cSERPINE2 was much higher in breast cancer tissues than in paired adjacent tissues in our cohort (Fig. 1c). Subsequently, we explored the relationship between cSERPINE2 expression and the clinicopathological characteristics of 136 breast cancer patients and found that cSERPINE2 might participate in the progression of breast cancer (Supplementary Table S1). Notably, breast cancer patients with larger tumor sizes and lymph node metastasis exhibited higher expression of cSERPINE2 (Fig. 1d, e). Moreover, Kaplan–Meier survival analysis revealed that breast cancer patients with higher cSERPINE2 expression exhibited shorter overall survival (OS) and recurrence-free survival (RFS) than those with lower cSERPINE2 expression (Fig. 1f). Additional Cox proportional hazards regression models suggested that upregulated cSERPINE2 expression was an independent prognostic indicator for OS and RFS in breast cancer patients (Supplementary Table S2 and S3).

cSERPINE2, derived from exon 2 and exon 3 of the SERPINE2 gene, formed a circular transcript of 509 nt (Fig. 1g). We investigated the expression of cSERPINE2 in breast cancer cell lines and normal breast cell line MCF-10A and found that cSERPINE2 was significantly upregulated in breast cancer cell lines (Supplementary Fig. S1a). For further characterization of cSERPINE2, RNase R treatment and half-life assay consistently showed that cSERPINE2 was much more stable than the linear transcript of SERPINE2 in MCF-7 and MDA-MB-468 cells (Supplementary Fig. S1b, c). Subsequent cell fractionation and fluorescent in situ hybridization (FISH) assays showed that cSERPINE2 was predominantly localized in the cytoplasm (Supplementary Fig. S1d, Fig. 1h). Collectively, these data suggest that cSERPINE2 is frequently upregulated in breast cancer, and it could serve as a potential indicator for unfavorable prognosis.

### EIF4A3 promoted the formation of cSERPINE2 in breast cancer

RNA-binding proteins (RBPs) participate in the biosynthesis of circRNA by binding to specific sequences to regulate the back splicing of circRNAs [25, 26]. To investigate which RBPs contributed to the formation of cSERPINE2, we chose 6 RBPs previously reported to participate in circRNA formation in cancers and detected the expression of cSERPINE2 after knocking down these RBPs (Supplementary Fig. S1e). Interestingly, we found that knocking down EIF4A3 significantly inhibited the expression of cSERPINE2 in MDA-MB-468 cells (Fig. 1i). Moreover, down-regulation of cSERPINE2 expression in EIF4A3 silenced cells could be restored by ectopic reintroduction of EIF4A3, indicating that EIF4A3 was involved in cSERPINE2 biogenesis (Supplementary Fig. S1f-h). EIF4A3 is a core component of the exon junction complex, which plays an essential role in pre-mRNA splicing [27]. To further explore the mechanisms by which EIF4A3 regulates cSERPINE2 biogenesis, we used CircInteractome to predict EIF4A3 binding sites and found two binding sites of EIF4A3 in the upstream flanking intron and across the self-exon 2 region of SERPINE2 pre-mRNA. Subsequently, we truncated the full length, upstream and downstream region of cSERPINE2 into four segments. RNA immunoprecipitation (RIP) assays indicated that EIF4A3 could bind the full-length sequence of cSERPINE2 (named “b”) and the upstream flanking sequence (named “d”) but not the other sites (“a” and “c”) (Fig. 1j). These results were further confirmed by RNA pull-down assays using *in vitro* transcript RNA fragments of the corresponding cSERPINE2 pre-mRNA (Fig. 1k).

In addition, the expression of cSERPINE2 in tumor tissues was positively related to the level of EIF4A3 in 27 clinical breast cancer specimens, suggesting that EIF4A3 was associated with cSERPINE2 expression (Fig. 1l). Furthermore, the expression of EIF4A3 was significantly increased in breast cancer tissues compared to normal tissues according to TCGA database analysis (Supplementary Fig. S1i). Kaplan–Meier survival analyses indicated that higher EIF4A3 expression was associated with poor OS and RFS in the breast cancer Kaplan–Meier plotter database (Supplementary Fig. S1j). Taken together, our findings suggest that EIF4A3 may facilitate the formation of cSERPINE2 by binding to flanking sequences.

### cSERPINE2 reshaped the immune microenvironment in breast cancer

**Fig. 2:**
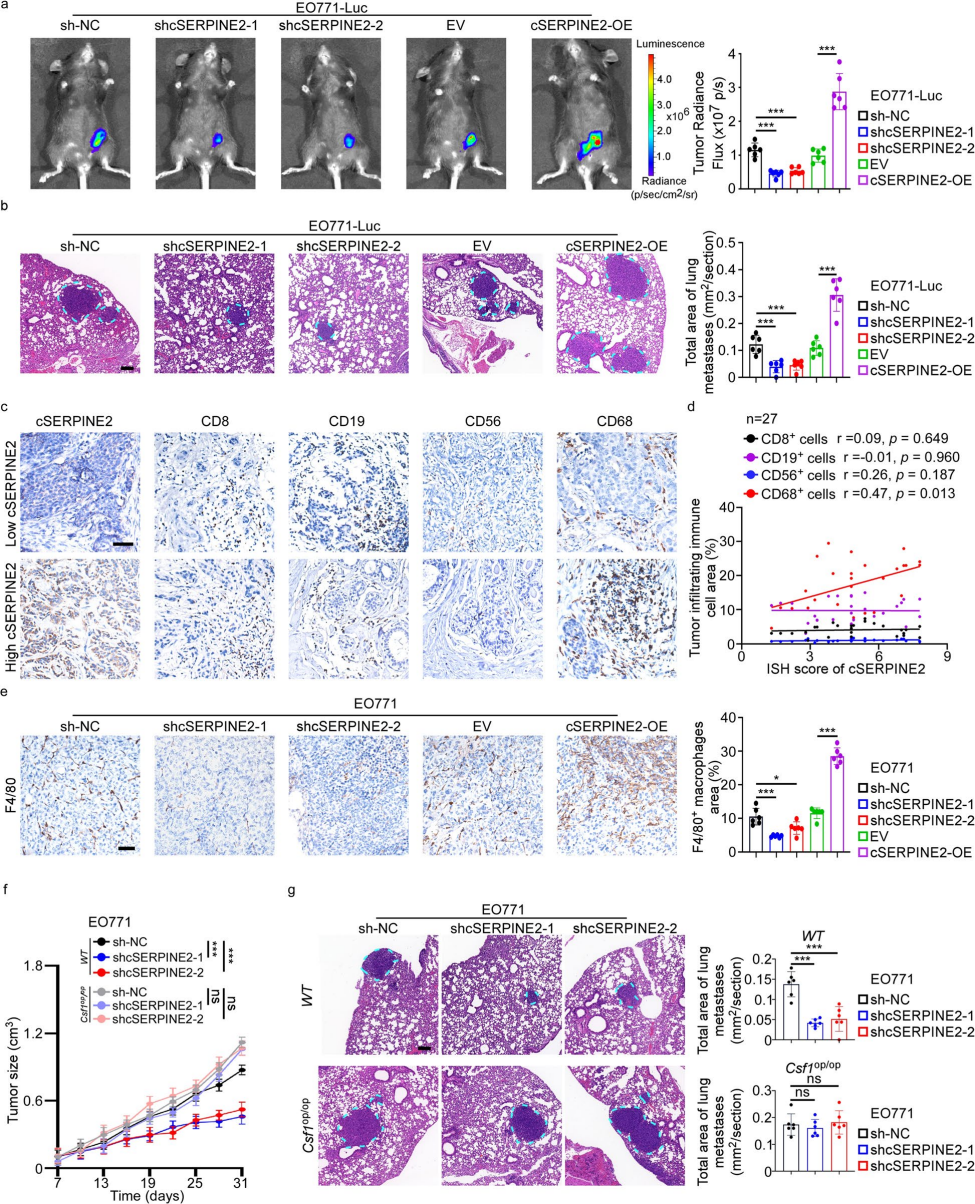
cSERPINE2 reshaped the immune microenvironment of breast cancer in mice. **a** Representative bioluminescence images (left) and quantification (right) of cSERPINE2 silenced and overexpressed tumor cells derived orthotopic breast cancer models (*n* = 6 mice for each group). **b** Representative lung H&E images (left) and quantification of lung metastatic area (right) of cSERPINE2 silenced and overexpressed tumor cells derived orthotopic breast cancer models (*n* = 6 mice for each group). Scale bar, 100 μm. **c** Representative IHC staining images of CD8^+^ T cells, CD19^+^ B cells, CD56^+^ NK cells and CD68^+^ macrophages in breast cancer tissues with low or high cSERPINE2 expression. Scale bar, 50 μm. **d** Correlation analysis of CD8^+^ T cells, CD19^+^ B cells, CD56^+^ NK cell and CD68^+^ macrophages infiltration and the cSERPINE2 expression in 27 breast cancer tissues. **e** Representative IHC staining images (left) and quantification (right) of F4/80^+^ macrophages infiltration in mice orthotopic breast cancer tissues. Scale bar, 50 μm. **f, g** cSERPINE2 silenced EO771 cells were used to constructed orthotopic tumor models in the wild-type (WT) or *csf1*^*op/op*^ mice (*n* = 6 for each group). Tumor size was measured over time (**f**). Representative H&E images (left) and quantification (right) of lung metastases (**g**). Scale bar, 100 μm. Data are presented as the means ± SD of three independent experiments. *P < 0.05, **P < 0.01, ***P < 0.001

To further investigate the biological functions of cSERPINE2 in breast cancer cells, we constructed stable cSERPINE2 knockdown cell lines using short hairpin RNAs that specifically targeted the back-splicing junction site of cSERPINE2. In addition, we also successfully constructed stable ectopic cSERPINE2 overexpression cell lines by transduction with the full-length cDNA of the cSERPINE2 lentiviral vector. The transduction efficiency was validated by qRT-PCR, but knocking down or overexpressing cSERPINE2 did not affect the level of SERPINE2 (Supplementary Fig. S2a). Unexpectedly, neither silencing nor overexpressing cSERPINE2 affected tumor cell proliferative and invasive capacity in vitro (Supplementary Fig. S2b, c). We observed high conservation between human and mouse cSERPINE2 through sequence alignment identification (Supplementary Fig. S2d). Therefore, we further explored the role of cSERPINE2 in vivo. Mouse cSERPINE2 was stably knocked down and overexpressed in EO771 cells which previously labeled with luciferase (Supplementary Fig. S2e), followed by orthotopic implantation into C57BL/6 mice. Contrary to in vitro results, tumor growth was significantly inhibited by cSERPINE2 silencing and accelerated by overexpression of cSERPINE2 (Fig. 2a). Furthermore, fewer formed lung metastases were detected following cSERPINE2 knockdown, while cSERPINE2 ectopic expression facilitated the occurrence of lung metastases (Fig. 2b). However, in vitro gain- and loss- of function assays showed that cSERPINE2 had no effect on the proliferative and invasive capacity of EO771 cells (Supplementary Fig. S2f, g). The substantial difference between cSERPINE2 function in vivo and in vitro indicated that cSERPINE2 might mainly influence the tumor immune microenvironment rather than the tumor cells themselves.

In an analysis of the association of CD45^+^ tumor-infiltrating leukocytes (CD8^+^ T cells, CD19^+^ B cells, CD56^+^ NK cells and CD68^+^ macrophages) and the cSERPINE2 level in breast cancer tissues (*n* = 27), immunohistochemistry (IHC) staining suggested that higher cSERPINE2 expression was notably associated with increasing CD68^+^ macrophages infiltration (Fig. 2c, d). Consistently, F4/80^+^ macrophages infiltration was elevated in the cSERPINE2-overexpressing xenograft tumors but decreased in the cSERPINE2-silenced xenograft tumors (Fig. 2e). To further investigate the role of macrophages in cSERPINE2-induced carcinogenesis, we constructed the *Csf1*^*op/op*^ mice that lack macrophages with mutated macrophage colony stimulating factor 1 (*Csf1*) and then constructed orthotopic tumor xenograft models [28]. As predicted, no significant differences in tumor size or lung metastasis formation were found in *Csf1*^*op*/*op*^ mice after transplantation of cSERINE2-silenced EO771 cells (Fig. 2f, g). Collectively, these results indicate that cSERPINE2 may promote breast cancer progression by increasing TAMs infiltration.

### Tumor exosomal cSERPINE2 targeted macrophages to promote the proliferation and invasion of breast cancer cells

**Fig. 3:**
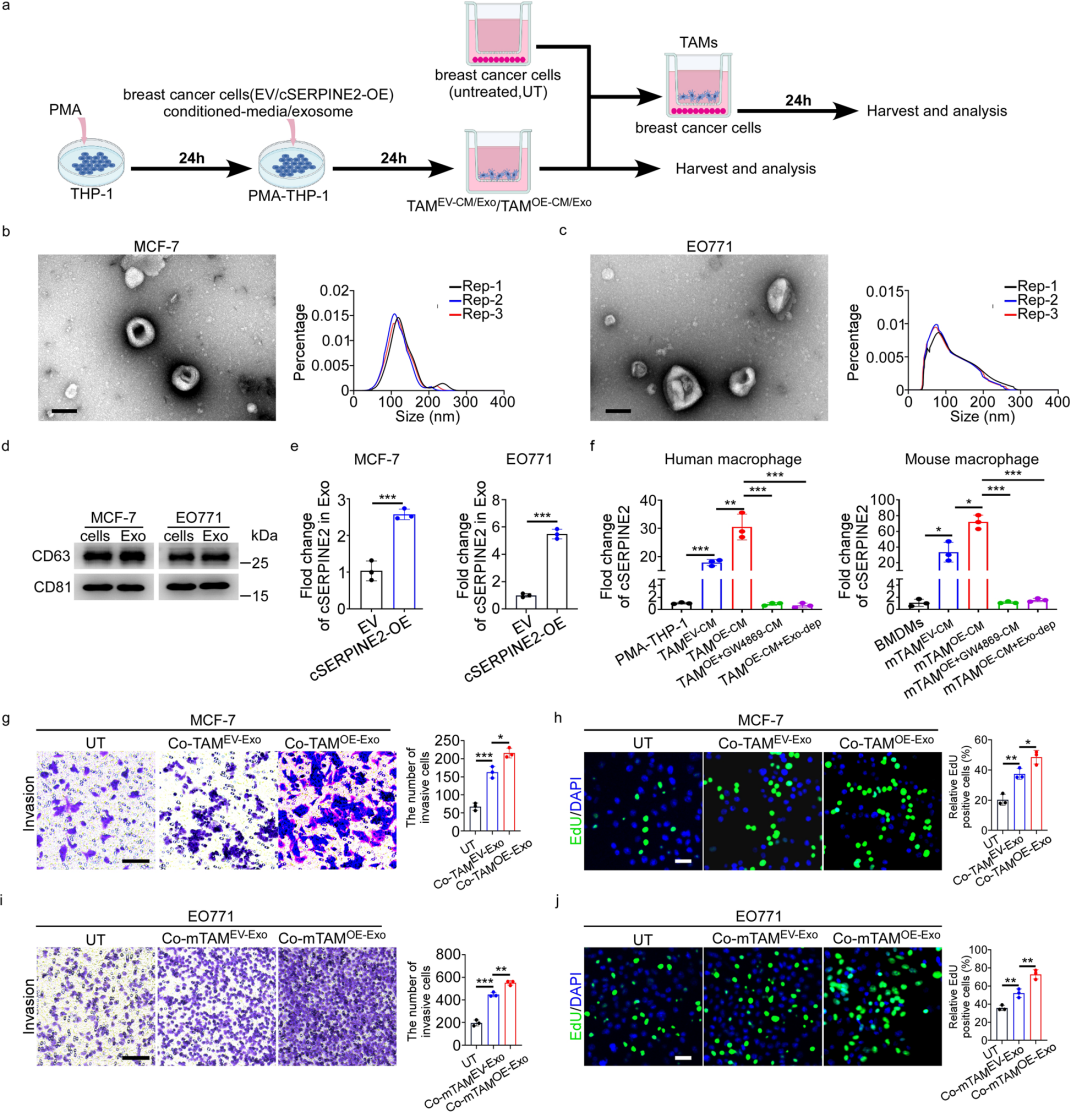
Tumor exosomal cSERPINE2 targeted macrophages to promote proliferation and metastasis of breast cancer cells. **a** THP-1 cells were treated with PMA, followed by the treatment of CM or exosomes derived from tumor cells as indicated treatments to obtain TAMs. Then tumor cells cocultured with these TAMs were used to subsequent experiments. **b**, **c** Phenotype analysis of exosomes derived from MCF-7 and EO771 cells using electron microscopy and NanoSight nanoparticle tracking analysis. Scale bar, 100 nm. **d** CD63 and CD81 expression in exosomes derived from MCF-7 and EO771 cells were detected by Western blotting. **e** The relative expression of cSERPINE2 in exosomes derived from MCF-7 and EO771 cells as indicated treatments. **f** The relative expression of cSERPINE2 in THP-1 or BMDM derived TAMs as indicated treatments. **g-j** The proliferation and invasion of MCF-7 and EO771 cells as indicated treatments in vitro were evaluated using EdU and transwell invasion assays, respectively. Scale bar, 20 μm. Data are presented as the means ± SD of three independent experiments. *P < 0.05, **P < 0.01, ***P < 0.001

To further elucidate the effects of tumor cSERPINE2 on TAMs, we induced THP-1 monocytic cells to differentiate into macrophages by stimulation with PMA for 24 h, and these THP-1-derived macrophages (PMA-THP-1) were further stimulated with cell-free conditioned media (CM) from MCF-7 cells transduced with empty vector or cSERPINE2 overexpression vector to obtain TAMs (TAM^EV−CM^ or TAM^OE−CM^) (Fig. 3a). Moreover, we isolated and differentiated murine bone marrow derived macrophages (BMDMs) from C57BL/6 mice, and then exposed them to cell-free CM collected from EO771 cells transduced with empty vector or cSERPINE2 overexpression vector to obtain BMDMs derived TAMs (mTAM^EV−CM^ or mTAM^OE−CM^). Consistent with previous studies [9, 29], THP-1 and BMDM-derived TAMs both exhibited a mixed M1/M2 phenotype, which not only showed higher levels of the M2 markers CD163 and CD206 but also increased the expression of proinflammatory genes, including TNF-α, IL-1β and IL-6 (Supplementary Fig. S3a, b). Since cSERPINE2 promoted breast cancer progression through TAMs in vivo, we constructed a coculture system in which tumor cells (MCF-7 and EO771) were cocultured with THP-1 and BMDM derived TAMs for 24 h, and subsequently, tumor cells were collected for EdU staining and transwell invasion assays (Fig. 3a). We found that the proliferative and invasive capacities of tumor cells were obviously enhanced after coculturing with TAMs, especially coculturing with TAM^OE−CM^, suggesting that TAMs promoted the proliferation and invasion of tumor cells and that this promotion effect was enhanced by cSERPINE2 overexpression in tumor cells (Supplementary Fig. S3c, d).

Previous studies have shown that tumor exosomal RNAs promoted tumor progression by regulating the interaction between tumor cells and TAMs [13, 30]. Hence, we speculated that cSERPINE2 could be shuttled from breast cancer cells to TAMs via exosomes and further educated TAMs, which impacted the proliferation and invasion of tumor cells. Therefore, we collected exosomes in CM from breast cancer cells using ultracentrifugation and the structure and size distribution of enriched exosomes were analyzed by transmission electron microscopy (TEM) and nanoparticle tracking analysis (Fig. 3b, c). Additionally, the exosomal markers CD63 and CD81 were detected on enriched exosomes (Fig. 3d). More importantly, the cSERPINE2 expression in enriched exosomes was positively related to the corresponding tumor cells, indicating that cSERPINE2 could be effectively packed into exosomes (Fig. 3e). Moreover, cSERPINE2 expression was significantly increased in TAMs, especially in TAM^OE−CM^ and mTAM^OE−CM^, suggesting that tumor exosomal cSERPINE2 could be internalized by macrophages (Fig. 3f). Furthermore, we inhibited exosome secretion of tumor cells by applying GW4869 or depleted exosomes from CM by ultracentrifugation, and found that the expression of cSERPINE2 was largely decreased in TAM^OE−CM^ or mTAM^OE−CM^ (Fig. 3f). These results indicated that cSERPINE2 could be encapsulated into tumor exosomes and further internalized by TAMs.

To further investigate the function of tumor exosomal cSERPINE2 internalized by TAMs, we stimulated PMA-THP-1 cells and BMDMs with exosomes from tumor cells transduced with empty vector or cSERPINE2 overexpression vector to acquire corresponding TAMs (TAM^EV/OE−Exo^ or mTAM^EV/OE−Exo^), respectively. Subsequently, tumor exosome-induced TAMs were cocultured with tumor cells for 24 h, and then, these tumor cells were collected for EdU staining and transwell invasion assays (Fig. 3a). Similar to the aforementioned results, the proliferation and invasion of MCF-7 and EO771 cells were notably increased after coculturing with exosome- pulsed TAMs, especially coculturing with TAM^OE−Exo^ and mTAM^OE−Exo^ (Fig. 3g-j). Taken together, our findings suggest that TAMs promote the proliferative and invasive behaviors of breast cancer cells by internalizing tumor exosomal cSERPINE2.

### Tumor exosomal cSERPINE2 upregulated MALT1 expression in macrophages by sponging miR-513a-5p

**Fig. 4:**
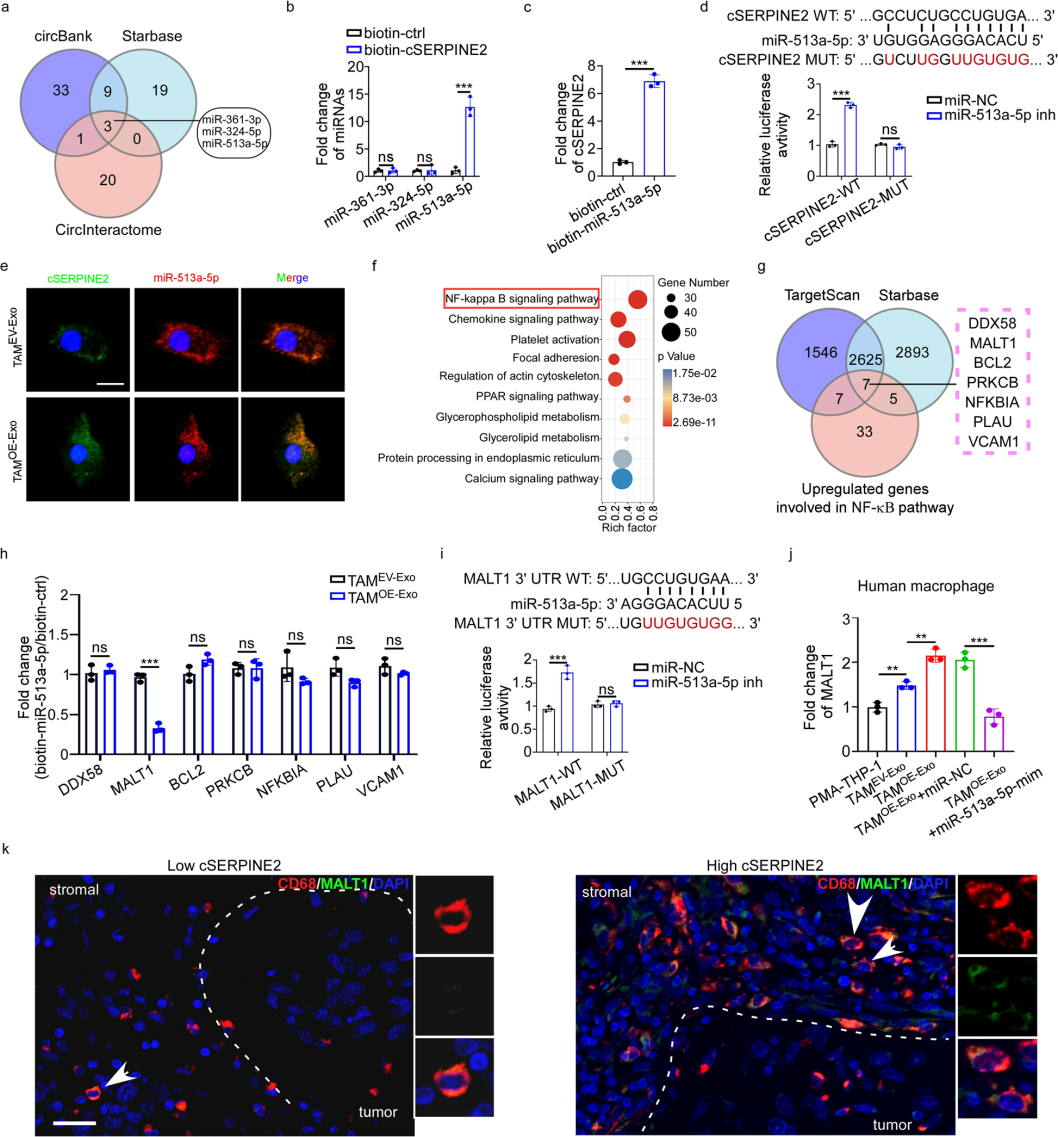
Tumor exosomal cSERPINE2 upregulated MALT1 expression in macrophages by sponging miR-513a-5p. **a** Identification of miRNAs that potentially bind to cSERPINE2 based on CircInteractome, circBank and Starbase databases. **b** RNA pull-down assay was performed in TAM^OE−Exo^ cells using biotinylated cSERPINE2-specific probe and control probe. The enrichment of miRNAs was detected by qRT-PCR. **c** RNA pull-down assay was performed in TAM^OE−Exo^ cells using biotinylated miR-513a-5p probe and control probe. The enrichment of cSERPINE2 was detected by qRT-PCR. **d** A schematic drawing showing the putative binding sites of miR-513a-5p in cSERPINE2 sequence, and the luciferase activity of cSERPINE2-WT or cSERPINE2-MUT in 293 T cells after co-transfection with miR-513a-5p inhibitor. **e** Representative FISH images of co-localization between miR-513a-5p and cSERPINE2 in TAM ^EV−Exo^ and TAM ^OE−Exo^ cells. Scale bar, 10 μm. **f** Pathway enrichment of the up-regulated genes in TAM^OE−Exo^ according to KEGG analysis. **g** Venn diagram showing the intersections of up-regulated genes involved in the NF-$\upkappa$B pathway and potential targets of miR-513a-5p predicted by Starbase and TargetScan. **h** RNA pull-down assay was performed in TAM^EV−Exo^ and TAM^OE−Exo^ cells using biotinylated miR-513a-5p probe and control probe. The enrichment of RNAs was detected by qRT-PCR. **i** A schematic drawing showing the putative binding sites of miR-513a-5p in MALT1 3’UTR sequence, and the luciferase activity of MALT1-WT or MALT1-MUT in 293 T cells after co-transfection with miR-513a-5p inhibitor. **j** The relative expression of MALT1 in PMA-THP-1 and TAMs as indicated treatments. **k** Immunofluorescence showing MALT1 expression (Green) on CD68^+^ macrophages (Red) in breast cancer tissues with low or high cSERPINE2 expression. Scale bar, 20 μm. Data are presented as the means ± SD of three independent experiments. *P < 0.05, **P < 0.01, ***P < 0.001

Since sponging miRNAs is a major mechanism of circRNAs [31], we analyzed the CircInteractome, circBank and StarBase online databases, and eventually identified 3 miRNAs as possible targets of cSERPINE2 (Fig. 4a). Moreover, we performed a circRNA pull-down assay to identify miRNAs interacted with cSERPINE2 in TAM^OE−Exo^ and found that miR-513a-5p could be pulled down by a biotin-labeled cSERPINE2 probe (Fig. 4b). Additionally, the miRNA pull-down assay further confirmed obvious enrichment of cSERPINE2 using biotin-miR-513a-5p in TAM^OE−Exo^ (Fig. 4c). Furthermore, we constructed the full-length cSERPINE2 sequence (cSERPINE2-WT) and cSERPINE2 sequences with mutant binding sites (cSERPINE2-MUT) followed by a luciferase reporter assay (Fig. 4d). Our results demonstrated that miR-513a-5p mimic and inhibitor significantly reduced and increased the luciferase activity of the luciferase reporter plasmid with cSERPINE2-WT respectively, while no change in the luciferase activity of the luciferase reporter plasmid with cSERPINE2-MUT was found (Fig. 4d and Supplementary Fig. S4a). Moreover, FISH assay suggested that cSERPINE2 colocalized with miR-513a-5p in the cytoplasm of TAM^EV−Exo^ and TAM^OE−Exo^ (Fig. 4e and Supplementary Fig. S4b). These results demonstrate that miR-513a-5p has a direct interaction with cSERPINE2 in TAMs.

To better understand the functions and mechanisms of cSERPINE2 in TAMs, we analyzed TAM transcriptomes by RNA-seq to compare differentially expressed genes (DEGs) between TAM^EV−Exo^ and TAM^OE−Exo^. Heatmap showed the top 200 upregulated and downregulated genes (Supplementary Fig. S4c). KEGG analysis showed that the upregulated genes were prominently enriched in the NF-$\upkappa$B pathway (Fig. 4f). Subsequently, we used StarBase and TargetScan to identify the potential target genes of miR-513a-5p and overlapped the potential target genes with the 52 upregulated genes involved in the NF-𝜅B pathway. Finally, 7 DEGs were identified as potential targets of the cSERPINE2/miR-513a-5p axis (Fig. 4g). Among the 7 DEGs, the miR-513a-5p in vivo pull-down assay revealed that the relative MALT1 enrichment bound by miR-513a-5p was prominently decreased in TAM^OE−Exo^ compared to TAM^EV−Exo^, suggesting that increased cSERPINE2 prevented miR-513a-5p binding to MALT1 (Fig. 4h). To further confirm that miR-513a-5p interacted with MALT1, we constructed a luciferase reporter gene containing the wild-type (WT) or mutant (MUT) MALT1 sequences. MiR-513a-5p mimics and inhibitor reduced and increased the luciferase activity, respectively, from 293 T cells transfected with MALT1-WT but not in MALT1-MUT group (Fig. 4i and Supplementary Fig. S4d). Furthermore, the expression of MALT1 was obviously upregulated in TAM^OE−Exo^ compared to TAM^EV−Exo^, and the upregulation of MALT1 was retarded by the miR-513a-5p mimic in TAM^OE−Exo^ cells (Fig. 4j).

The paracaspase MALT1, a key regulator of immune responses, could induce a component of the signaling pathway mediating antigen receptor-dependent stimulation of the transcription factor NF-𝜅B [32, 33]. Although abnormal MALT1 expression is closely associated with lyphomagenesis and antoimmune diseases, the function of MALT1 in breast cancer is still unclear [34–36]. MALT1 expression has been noted in several types of cancer, with low expression levels in breast cancer indexed in the Human Protein Atlas database (Supplementary Fig. S4e). In our cohorts of breast cancer patients, MALT1 expression was barely present in tumor cells but was enriched in CD68^+^ TAMs in breast cancer tissues with high expression cSERPINE2 (Fig. 4k and Supplementary Fig. S4f). Moreover, enforced cSERPINE2 expression had no effect on MALT1 expression in MCF-7 and EO771 cells, but obviously promoted MALT1 expression in PMA-THP-1 and BMDMs cells (Supplementary Fig. S4g-h). Collectively, these results suggest that the MALT1 levels are regulated by tumor exosomal cSERPINE2 by sponging miR-513a-5p in TAMs, while this regulatory mechanism of MALT1 is not present in breast cancer cells.

### Tumor exosomal cSERPINE2 promoted IL-6 secretion of TAMs through activating the NF-𝜅B pathway to enhance breast cancer progression

**Fig. 5:**
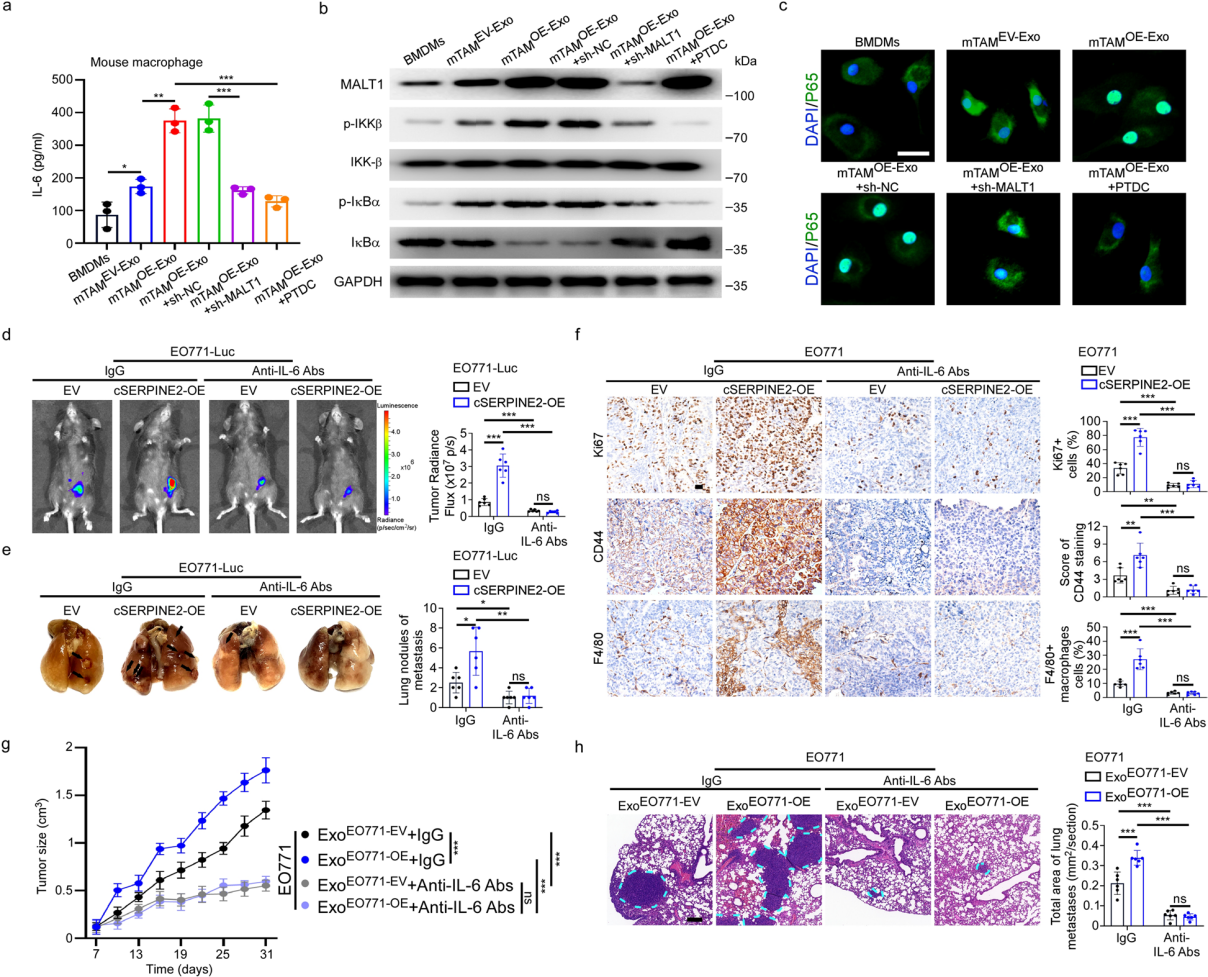
Tumor exosomal cSERPINE2 promoted the secretion of IL-6 in TAMs via activating the NF-$\upkappa$B pathway to encourage progression of breast cancer. **a** ELISA assay of IL-6 concentration in the supernatants from BMDMs and mTAMs as indicated treatments. **b** Western blotting was performed to evaluate the expression levels of MALT1, phosphorylated IKK-β, IKK-β, phosphorylated IκBα and IκBα in BMDMs and mTAMs as indicated treatments. **c** p65 nuclear translocation in BMDMs and mTAMs as indicated treatments was assessed by immunofluorescent confocal microscopy. Scale bar, 5 μm. **d** Representative bioluminescence images (left) and quantification (right) of cSERPINE2 silenced and overexpressed tumor cells derived orthotopic breast cancer model after IgG or anti-IL-6 Abs treatments (*n* = 6 mice for each group). **e** Representative lung images (left) and quantification of metastatic foci (right) of cSERPINE2 silenced and overexpressed tumor cells derived orthotopic breast cancer model after IgG or anti-IL-6 Abs treatments (*n* = 6 mice for each group). **f** Representative IHC images (left) and quantification (right) of Ki67, CD44 expression and F4/80^+^ macrophages infiltration in cSERPINE2 silenced and overexpressed tumor cells derived orthotopic breast cancer model after IgG or anti-IL-6 Abs treatments. Scale bar, 50 μm. **g, h** Orthotopic breast cancer models were injected exosomes derived from EO771 cells with or without cSERPINE2 overexpression, followed by treatment of IgG or anti-IL-6 antibody (*n* = 6 for each group). Tumor size was measured over time (**g**). Representative H&E images (left) and quantification (right) of lung metastases (**h**). Scale bar, 100 μm. Data are presented as the means ± SD of three independent experiments. *P < 0.05, **P < 0.01, ***P < 0.001

We observed that the IL-6 expression was substantially upregulated in TAM^OE−CM^ compared to TAM^EV−CM^, indicating that the expression of IL-6 in TAMs may be regulated by tumor exosomal cSERPINE2 (Supplementary Fig. S3a, b). Indeed, ELISA results confirmed that the level of IL-6 was significantly increased in the supernatants of TAM^OE−Exo^ compared to TAM^EV−Exo^ (Supplementary Fig. S5a). Since MALT1 promoted IL-6 secretion by activating the NF-$\upkappa$B pathway in macrophages, we asked whether tumor exosomal cSERPINE2 promoted IL-6 secretion of TAMs through activation of the NF-𝜅B pathway [37, 38]. As expected, silencing MALT1 or the additional treatment with PDTC (NF-κB inhibitor) in TAM^OE−Exo^ inhibited the increasing IL-6 secretion (Supplementary Fig. S5a). Furthermore, western blotting indicated that the levels of phosphorylated I𝜅Bα and IKK-β were notably increased in TAM^OE−Exo^, while these effects were retarded by MALT1 knockdown or the addition of PDTC treatment (Supplementary Fig. S5b-c). Similar findings were obtained in mTAMs using the above mentioned assays and western blotting quantification analysis (Fig. 5a-b and Supplementary Fig. S5d). Moreover, p65 nuclear translocation was notably increased in mTAM^OE−Exo^, while silencing MALT1 or adding PDTC treatment significantly inhibited p65 nuclear translocation (Fig. 5c). These results suggested that tumor exosomal cSERPINE2 promoted the IL-6 expression by upregulating MALT1 and activating the NF-$\upkappa$B pathway in TAMs.

Cytokine secretion represents the major functional response of macrophages [8]. Next, we asked whether the increased IL-6 levels were responsible for the tumor-promoting function of tumor exosomal cSERPINE2. Breast cancer orthotopic xenograft model was constructed with EO771 cells transduced with empty vector or cSERPINE2 overexpression vector followed by intraperitoneal injection of IgG or anti-IL-6 antibody. Surprisingly, the promoting effect of cSERPINE2 overexpression on tumor growth and metastasis was blocked by anti-IL-6 antibody (Fig. 5d, e). Furthermore, IHC staining revealed that the increased expression of Ki67 and CD44 (invasive marker) and the elevated infiltration of F4/80^+^ macrophages in cSERPINE2 overexpressing tumor tissues were blocked by anti-IL-6 antibody (Fig. 5f). In particular, we injected exosomes enriched from EO771 cells transduced with empty vector or cSERPINE2 overexpression vector (Exo^EO771−EV^ or Exo^EO771−OE^) followed by injection of IgG or anti-IL-6 antibody in orthotopic xenograft models. Notably, the tumor size and lung metastatic formation largely increased after injection of Exo^EO771−OE^ in the IgG group, while no significant difference was observed after injection of anti-IL-6 antibody (Fig. 5g, h). Additionally, the expression of Ki67 and CD44 and the infiltration of F4/80^+^ macrophages were not affected by treatment of Exo^EO771−OE^ in the anti-IL-6 antibody group (Supplementary Fig. S5e).

IL-6 usually binds to its receptor complex IL6R/gp130 and activates downstream Janus kinases (JAKs), which subsequently activate STAT3 through phosphorylation of tyrosine 705 [39]. Unsurprisingly, analysis of harvested xenograft tumors showed that the levels of p-JAK2 and p-STAT3 notably increased after injection of Exo^EO771−OE^ in the IgG group, while no significant changes were observed in the anti-IL-6 antibody group (Supplementary Fig. S5f-g). Consistently, the levels of p-JAK2 and p-STAT3 notably increased in EO771 cells after coculturing with mTAM^OE−Exo^ in contrast to mTAM^EV−Exo^ followed by IgG treatment, whereas anti-IL-6 antibody abrogated these effects (Supplementary Fig. S5h-i). Together, these results demonstrate that tumor exosomal cSERPINE2 promotes the secretion of IL-6 in TAMs by activating the NF-$\upkappa$B pathway to encourage breast cancer progression.

### IL-6 promoted EIF4A3 and CCL2 levels in tumor cells by activating the JAK2-STAT3 pathway

**Fig. 6:**
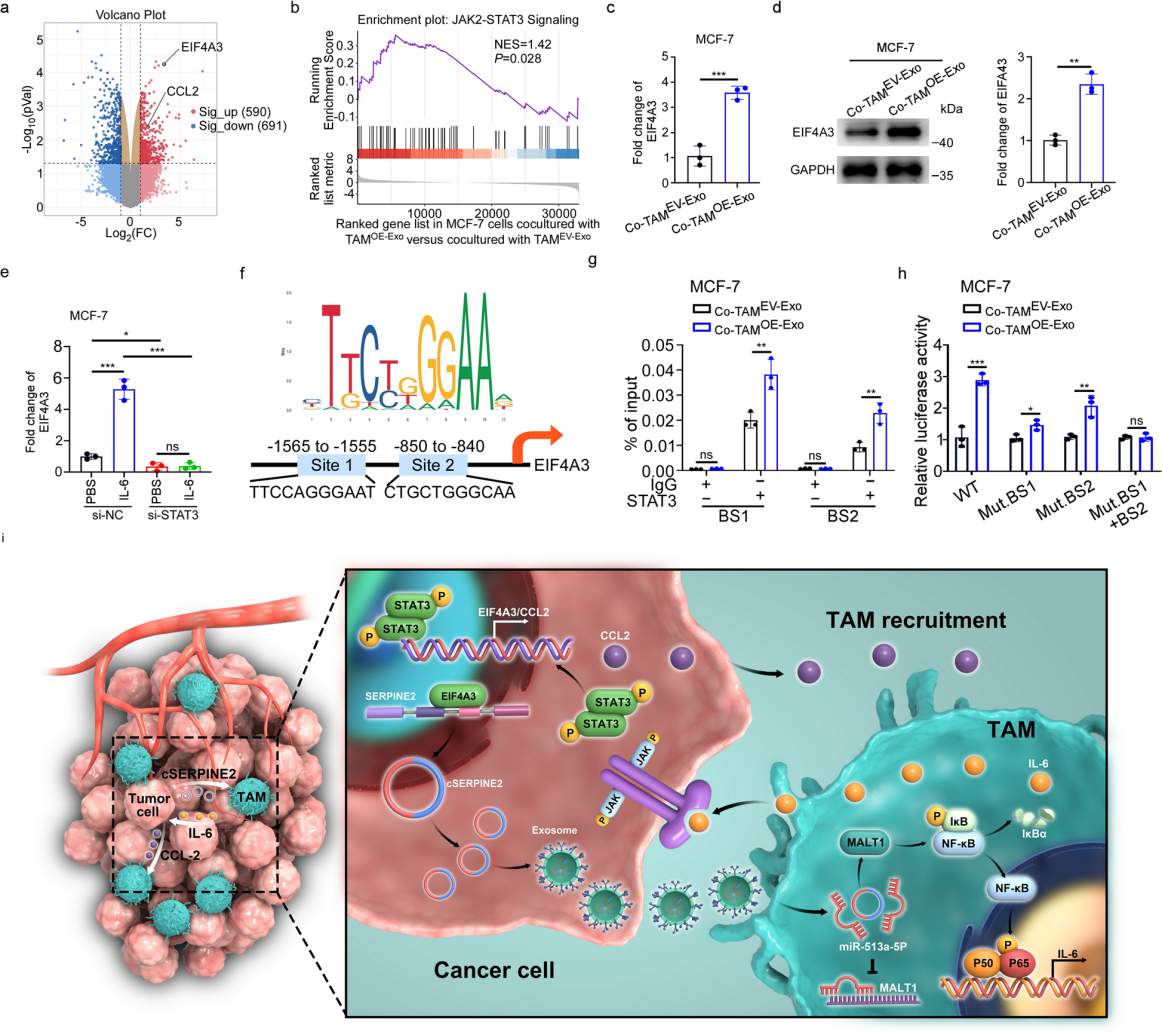
IL-6 promoted the expression of EIF4A3 in tumor cells via activating JAK2-STAT3 pathway**. a** Volcano plot showing differentially expressed genes in MCF-7 cells co-cultured with TAM^OE−Exo^ compared to co-cultured with TAM^EV−Exo^. **b** GSEA showing significantly upregulated genes in MCF-7 cells co-cultured with TAM^OE−Exo^ were significantly enrichment in JAK2-STAT3 pathway. **c** EIF4A3 expression in MCF-7 cells co-cultured with TAM^EV−Exo^ or TAM^OE−Exo^ was determined by qRT-PCR. **d**. Western blotting analysis (left) and quantification (right) of EIF4A3 level in MCF-7 cells co-cultured with TAM^EV−Exo^ or TAM^OE−Exo^. **e** The relative expression of EIF4A3 in MCF-7 cells as indicated treatments. **f** Sequence motif representing the consensus STAT3 binding motif (JASPAR database, upper) and schematic diagram of the putative STAT3 binding site in the EIF4A3 promoter (lower). **g** ChIP assay was performed in MCF-7 cells co-cultured with TAM^EV−Exo^ or TAM^OE−Exo^ to detect the enrichment of potential binding sequences using the STAT3 antibody. **h** Luciferase reporter assay was performed to validate the interaction between STAT3 and the two binding sites (BS1 and BS2). **i** Schematic illustrating the molecular mechanism of tumor exosomal cSERPINE2 triggered a positive feedback loop between tumor cells and TAMs to promote breast cancer progression. Data are presented as the means ± SD of three independent experiments. *P < 0.05, **P < 0.01, ***P < 0.001

To better uncover the mechanism of IL-6/STAT3 signaling in TAM-educated tumor cells, we performed RNA-seq on MCF-7 cells after coculturing with TAM^EV−Exo^ or TAM^OE−Exo^. Transcriptional profiling revealed that 590 genes were upregulated and 691 genes were downregulated (|log_2_FC|≥ 1; FDR ≤ 0.05) (Fig. 6a). More interestingly, we found that EIF4A3 and CCL2 were prominently upregulated in MCF-7 cells educated with TAM^OE−Exo^ compared to those educated with TAM^EV−Exo^. Gene set enrichment analysis (GSEA) analysis confirmed that the JAK2-STAT3 pathway was enriched as the top pathway in MCF-7 cells after coculturing with TAM^OE−Exo^ (Fig. 6b).

As described above, the RNA binding protein EIF4A3 is considered as an important regulator of post-transcriptional regulatory processes [40], and CCL2, as a powerful macrophage chemokine, was reported to be a target of STAT3 in breast cancer [41]. Here, we further confirmed that the EIF4A3 and CCL2 levels were notably increased in MCF-7 cells after coculturing with TAM^OE−Exo^ (Fig. 6c, d and Supplementary Fig. S6a). Nevertheless, ectopic expression of cSERPINE2 in MCF-7 cells did not regulate the expression of EIF4A3 or CCL2 or activation of the JAK2-STAT3 pathway, which indirectly indicated that cSERPINE2 overexpression did not affect tumor cell proliferative and invasive capacity in vitro (Supplementary Fig. S6b-d). Consistent with the above data, silencing STAT3 in the MCF-7 cells stimulated with IL-6 led to striking downregulation of EIF4A3 and CCL2, suggesting that STAT3 participated in the regulation of EIF4A3 and CCL2 (Fig. 6e and Supplementary Fig. S6e-f). By investigating the mechanisms by which STAT3 regulates EIF4A3 and CCL2 expression using the Jaspar database, we firstly found two binding sites (BS1/BS2) for STAT3 in the promoter of EIF4A3 (Fig. 6f). ChIP-qPCR revealed that MCF-7 cells cocultured with TAM^OE−Exo^ had significantly increased STAT3 occupancies on the BS1 and BS2 of the promoter of EIF4A3 (Fig. 6g). Furthermore, a luciferase plasmid with the top 2000 nucleotides of the promoter domain of the EIF4A3 gene (EIF4A3-WT) and a luciferase plasmid with mutant sequences in BS1 or/and BS2 of the promoter domain (EIF4A3-BS1/BS2-MUT) were generated and transfected into MCF-7 cells. Luciferase reporter assays revealed that the TAM^OE−Exo^ induced MCF-7 cells enhanced the luciferase activity of EIF4A3-WT, but mutation in either STAT3 binding site partially abrogated this promotion and mutations in both sites completely abrogated it (Fig. 6h). Similar to the results of EIF4A3, two binding sites (BS1/BS2) for STAT3 in the promoter of CCL2 were predicted in the Jaspar database (Supplementary Fig. S6g). ChIP-qPCR suggested that the STAT3 occupancies on the both BS1 and BS2 of the promoter of CCL2 were significantly increased in the TAM^OE−Exo^ induced MCF-7 cells (Supplementary Fig. S6h). Moreover, our results indicated that the supernatant from the MCF-7 cells cocultured with TAM^OE−Exo^ significantly promoted PMA-THP-1 migration compared to that of supernatant from the tumor cells cocultured with TAM^EV−Exo^ (Supplementary Fig. S6i-j).

We summarized our findings in a schematic (Fig. 6i). Briefly, breast cancer cells exosomal cSERPINE2 was shuttled to TAMs and notably elevated MALT1 levels, enhancing the secretion of IL-6 by activating the NF-$\upkappa$B pathway and leading to increased proliferation and invasion of breast cancer cells. More importantly, IL-6 in turn increased the EIF4A3 and CCL2 levels within tumor cells in a positive feedback mechanism, further enhancing tumor cSERPINE2 biogenesis and promoting the recruitment of TAMs.

### Characteristics of PLGA-PEG (si-cSERPINE2) NPs

**Fig. 7:**
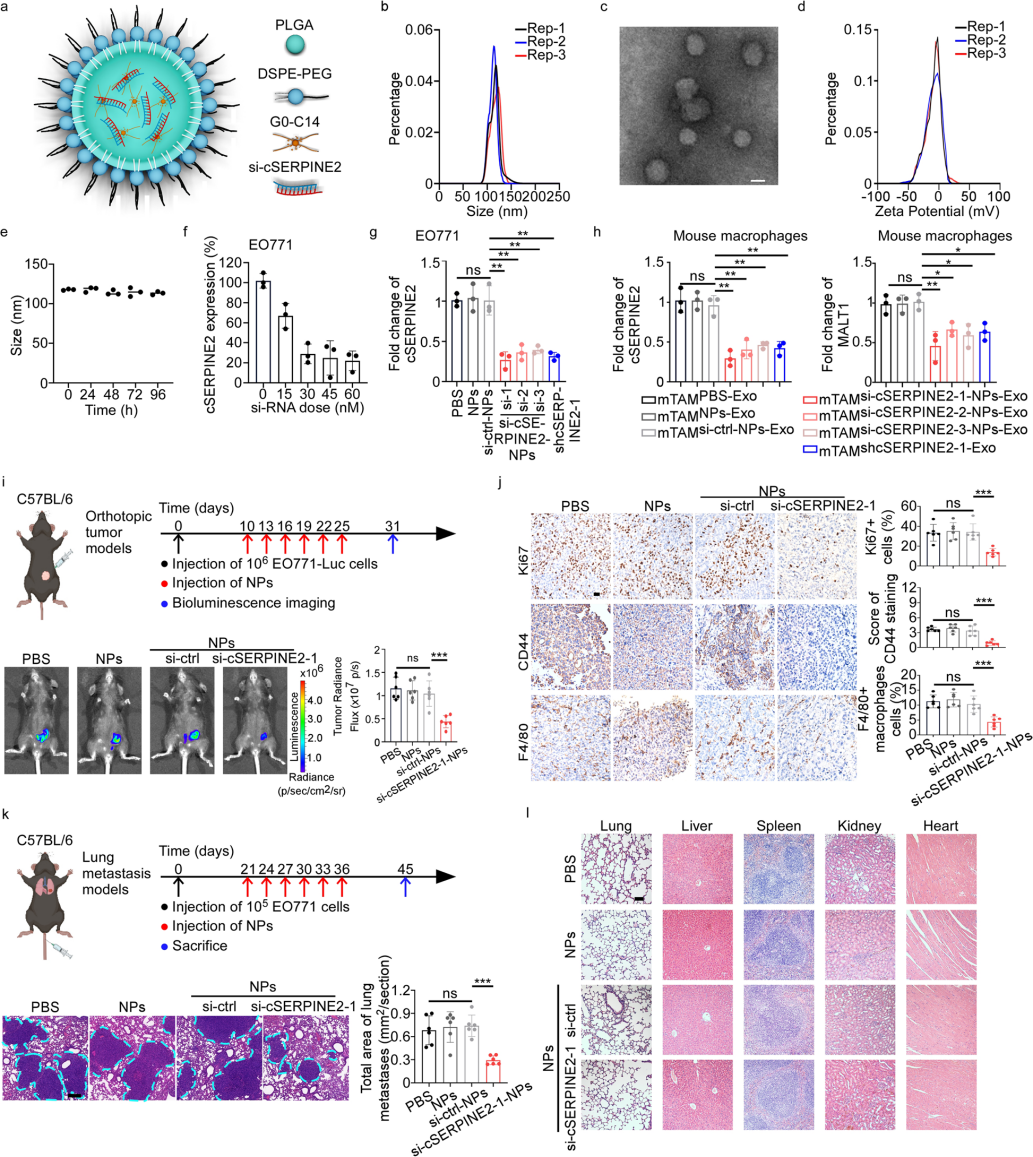
Characteristics of si-cSERPINE2-NPs and therapeutic efficacy of si-cSERPINE2-NPs in breast cancer in vivo. **a** Schematic illustration of the si-cSERPINE2-NPs. **b** Size distribution of the si-cSERPINE2-NPs in the aqueous solution detected by Dynamic light scattering (DLS). **c** Representative TEM images of si-cSERPINE2-NPs. Scale bar, 100 nm. **d** Zeta potential of si-cSERPINE2-NPs in the aqueous solution detected by DLS. **e** Stability of si-cSERPINE2-NPs in 10% serum at 37 °C was evaluated by measuring particle size changes with DLS at various time points up to 96 h. **f** The relative expression of cSERPINE2 in EO771 cells treated with si-cSERPINE2-NPs at different siRNA doses. **g** The relative expression of cSERPINE2 in EO771 cells treated with PBS, NPs, si-ctrl NPs, si-cSERPINE2-NPs or shcSERPINE2-1. **h** The relative expression of cSERPINE2 (left) and MALT1 (right) in mTAMs as indicated treatments. **i** Schematic illustration (upper) of tumor inoculation and different treatments in EO771-Luc orthotopic breast cancer models. Representative bioluminescence images and quantification (lower) of the EO771-Luc orthotopic breast cancer models. **j** IHC staining of Ki67, CD44 expression and F4/80^+^ macrophages infiltration in the tumor tissues of orthotopic breast cancer models. scale bar, 20 μm. **k** Schematic illustration (upper) of tumor inoculation and different treatments in EO771 lung metastatic models. Representative H&E images (left) and quantification (right) of lung metastases. Scale bar, 100 μm. **l** Histological section of the major organs after six consecutive injections of PBS, NPs, si-ctrl NPs or si-cSERPINE2-1-NPs in EO771-luc orthotopic breast cancer models. Scale bar, 20 μm. Data are presented as the means ± SD of three independent experiments. *P < 0.05, **P < 0.01, ***P < 0.001

To investigate the potential utility of cSERPINE2 as a therapeutic target in breast cancer, we applied a self-assembly strategy for formulating lipid-polymer hybrid NPs for systemic si-cSERPINE2 delivery, composed of the ionizable lipid-like compound G0-C14 for si-cSERPINE2 complexion, a biocompatible poly lactic-co-glycolic acid (PLGA) polymer to form a stable NP core to carry the G0-C14/si-cSERPINE2 and a lipid-poly (ethylene glycol) (lipid-PEG) layer for stability (Fig. 7a). The si-cSERPINE2 loaded NPs were ~ 114.63 nm in size, as measured by dynamic light scattering (DLS) (Fig. 7b). Their morphology was assessed by transmission electron microscopy and the zeta potential was ~ -9.09 mV (Fig. 7c, d). Moreover, there were no obvious changes in the size of si-cSERPINE2-NPs over a period of 96 h in the presence of 10% serum, indicating acceptable stability of these engineered NPs (Fig. 7e). Furthermore, the si-cSERPINE2-NPs efficiently silenced cSERPINE2 expression in tumor cells and more than 70% of cSERPINE2 expression could be inhibited at a siRNA dose of 30 nM (Fig. 7f). With a siRNA dose of 30 nM, si-cSERPINE2-NPs significantly silenced cSERPINE2 expression in tumor cells compared to si-ctrl-NPs (Fig. 7g and Supplementary Fig. S7a). We next examined whether si-cSERPINE2-NPs treatment of tumor cells could influence the cSERPINE2 and MALT1 levels in TAMs. As mentioned in the aforementioned assays, we found that cSERPINE2 and MALT1 expression was significantly decreased in the TAMs educated by exosomes of the tumor cells pretreated with si-cSERPINE2-NPs (TAM ^si−cSERPINE2−NPs−Exo^) compared to TAMs educated by exosomes of the tumor cells pretreated with si-ctrl-NPs (TAM^si−ctrl−NPs−Exo^) (Fig. 7h and Supplementary Fig. S7b-d). Moreover, the proliferative and invasive ability of tumor cells were obviously repressed after coculturing with TAM ^si−cSERPINE2−NPs−Exo^ compared to coculture with TAM^si−ctrl−NPs−Exo^ (Supplementary Fig. S7e-h).

To further evaluate the properties of si-cSERPINE2 NP delivery in vivo, we first conducted pharmacokinetics studies by injecting cy5-si-cSERPINE2-1-NPs or naked-cy5-si-cSERPINE2-1 into healthy C57BL/6 mice through the tail vein, and the results showed that the cy5-si-cSERPINE2-1-NPs had prolonged siRNA circulation (Supplementary Fig. S7i). Next, we investigated the biodistribution and tumor accumulation of these NPs in orthotopically incubated EO771 tumor-bearing mice. As shown in Supplementary Fig. S7j, cy5-si-cSERPINE2-1-NPs exhibited higher tumor accumulation. With the promising in vitro results, prolonged blood circulation and high tumor accumulation described above, the effects of si-cSERPINE2-NPs on breast cancer in vivo are promising.

### Therapeutic efficacy and toxicity evaluation of systemic injection of PLGA-PEG (si-cSERPINE2) NPs in breast cancer

To better illustrate the potential therapeutic effect of si-cSERPINE2-NPs on breast cancer *in vivo*, we randomly divided the tumor-bearing mice into four groups. The mice in the PBS, control NPs, si-ctrl NPs and si-cSERPINE2-1-NPs groups were injected via the tail vein with a siRNA dose of 700 μg/kg body weight every 3 days for 6 cycles after average tumor size had increased to ~ 120mm^3^ (about day 10) (Fig. 7i). At day 31, all mice were euthanized and we found that si-cSERPINE2-1-NPs obviously suppressed tumor growth (Fig. 7i). qRT-PCR results confirmed the consistent knockdown of cSERPINE2 in tumor tissues derived from the mice treated with si-cSERPINE2-1-NPs (Supplementary Fig. S8a). In addition, IHC staining showed that si-cSERPINE2-NPs treatment inhibited the expression of Ki67 and CD44 and the infiltration of F4/80^+^ macrophages (Fig. 7j).

Furthermore, we generated lung metastasis models to explore the effect of si-cSERPINE2-1-NPs in regulating the tumor metastasis *in vivo.* After 3 weeks of tail vein injection of EO771 cells, PBS, NPs, si-control NPs or si-cSERPINE2-1-NPs were injected into mice via tail vein every 3 days for 6 cycles (Fig. 7k). Surprisingly, we found that the lung metastatic tumor burden was significantly reduced in the si-cSERPINE2-1-NPs group compared to other groups (Fig. 7k). Regardless of the orthotopic tumor model or lung metastasis model mice, no remarkable changes in mouse body weight were found during the whole treatment period, indicating no apparent systemic toxicity for any treatment group (Supplementary Fig. S8b). Furthermore, the results of systemic toxicity revealed that intravenous administration of si-cSERPINE2-1-NPs exhibited no significant toxicity to major organs, and blood index analyses confirmed the absence of significant hepatotoxicity and renotoxicity (Fig. 7l and Supplementary Fig. S8c).

## Discussion

During tumor progression and metastasis, complex communication networks between tumor cells and stromal cells determine the effect of clinical intervention [42, 43]. TAMs are the most prominent stromal cells that inhibit the anti-tumor immune response [8, 44]. Previous reports found that circRNAs enable TAMs to interact with tumor cells or other stromal cells by secreting cytokines and chemokines to modulate tumor progression [45]. However, the role of tumor derived circRNAs in TAMs remains largely unclear.

A variety of evidence showed that the extracellular serine protease inhibitor SERPINE2 fosters the metastasis of breast cancer cells by remodeling the extracellular matrix [19, 20, 46, 47]. Moreover, the secreted protein SERPINE2 not only drives the formation of extravascular networks but also ensures cancer cell perfusion by acting as an anticoagulant, finally leading to distant metastasis of breast cancer [20]. Hence, SERPINE2 is regarded as a driver of metastatic progression in human cancer. Nevertheless, the current explorations of SERPINE2 cannot fully illustrate the diversity of its involvement in tumor development, especially, the function of circRNAs arising from SERPINE2 has not been fully elucidated. In this study, we revealed a novel mechanism by which cSERPINE2 function as a communication mediator in the TME. cSERPINE2 exhibited increased expression in breast cancer and was closely associated with poor survival. Interestingly, cSERPINE2 did not directly promote the malignant phenotype of breast cancer cells in vitro, while high cSERPINE2 expression increased the infiltration of TAMs and drove breast cancer progression in vivo. Mechanistically, tumor exosomal cSERPINE2 was shuttled to TAMs and notably elevated MALT1 levels, which enhanced the secretion of IL-6 through activating the NF-$\upkappa$B pathway, leading to increased proliferation and invasion of breast cancer cells.

The paracaspase MALT1 functions as a central role in the activation of immune cells by transducing NF-$\upkappa$B signaling [32, 33]. NF-$\upkappa$B targeted genes, which encoding cytokines and antiapoptotic proteins, promote the efficient generation of immune response [48]. Recent studies have indicated that MALT1 inhibition impairs immune suppressive function of regulatory T cell in the TME, indicating that MALT1 may suppress anti-tumor immunity in solid cancers [49]. Notably, MALT1 drives epithelial-to-mesenchymal transition (EMT) in claudin-low, triple-negative breast cancer (TNBC) with overexpression of selected G protein-coupled receptors (GPCRs) [50], suggesting MALT1 as a novel oncogenic signaling molecule in a subset of GPCR^+^TNBCs. Here, we found that MALT1 is barely expressed on tumor cells but is widely expressed on TAMs in breast cancer patients with high expression of cSERPINE2. Indeed, breast cancer cells notably elevated MALT1 expression in TAMs via tumor exosomal cSERPINE2, which further activated the NF-$\upkappa$B pathway and promoted the secretion of IL-6. However, the level of MALT1 and the activation of the NF-$\upkappa$B pathway were not determined by cSERPINE2 in breast cancer cells. This “spatially isolated” MALT1 expression and its regulation may contribute to breast cancer immune evasion and promote tumor progression. Similarly, the “spatially isolated” CD39-CD73 expression was observed in hepatocellular carcinoma (HCC) environment, which CD39^+^ macrophages surrounded by CD73^+^ HCC cells and finally lead to poor response to the PD1 antibody in HCC [13]. In fact, the spatial differences of gene expression across different regions of tumor were known as intratumor heterogeneity [51]. Recently, studies suggest that induction of the hypoxia-responsive gene expression program is accompanied by spatial alterations of genome, which might explain why existed “spatially isolated” of gene expression [51, 52].

IL-6, one of the major cytokines in the TME, promotes tumorigenesis by regulating the hallmarks of cancer and multiple signaling pathways [53–55]. Our findings demonstrated that tumor exosomal cSERPINE2 increased IL-6 levels by activating the NF-$\upkappa$B pathway of TAMs and that blocking IL-6 reversed the tumor-promoting effect of cSERPINE2 overexpression in vivo. Additionally, TAM-derived IL-6 in turn elevated CCL2 and EIF4A3 expression in tumor cells by increasing the activation of the JAK2-STAT3 pathway. Similar to previous studies, our study found that increased IL-6 secretion by tumor exosomal cSERPINE2-educated TAMs elevated the expression of CCL2 to enhance TAMs infiltration [41]. Moreover, the increased EIF4A3 expression in TAMs educated tumor cells promoted the biosynthesis of cSERPINE2. Herein, our study disclosed a novel positive feedback loop between TAMs and cancer cells that is essential to the proliferation and metastasis of breast cancer, implying that the signal cascade initiated by cSERPINE2 might be a potential therapeutic target.

PLGA was characterized by biodegradability, non-toxicity and high stability, suggesting that PLGA-based NPs incorporating siRNA may serve as a promising nanotherapeutic strategy for the treatment of cancers [56–59]. In our study, we developed and characterized a PEGylated PLGA nanoplatform loaded with si-cSERPINE2 for breast cancer therapy. The PLGA-based nanosystem protected si-cSERPINE2 from premature degradation. The enhanced permeability and retention (EPR) effect is an interesting concept in which nanoparticles of certain sizes tend to accumulate in tumor tissues due to the leaky vasculature and poor lymphatic drainage present in the tumor. Taking advantage of the EPR effect, PLGA-PEG (si-cSERPINE2) NPs achieved high concentrations in breast cancer xenografts in vivo. Moreover, the results from breast orthotopic and lung metastatic models showed that this si-cSERPINE2-NPs system prominently reduced tumor burden and lung metastatic formation. Additionally, the results from H&E histopathological analysis and blood biochemical examination confirmed si-cSERPINE2-NPs had not significant toxic side effects. Hence, si-cSERPINE2-NPs with high specificity and in vivo safety can be explored to enable therapeutic gain for breast cancer patients.

## Conclusion

In this work, we unraveled a novel mechanism that oncogene SERPINE2-derived hsa_circ_0001103 (cSERPINE2) functioned as communication signaling in tumor immune microenvironment to promote breast cancer progression. Different from cognate gene SERPINE2, overexpression of cSERPINE2 cannot encourage the malignant phenotype of breast cancer cells in vitro. Interestingly, enforced cSERPINE2 expression increased TAMs infiltration and further drove breast cancer progression in vivo, while these effects were abolished in *csf1*^*op/op*^ mice. Furthermore, we uncovered a positive feedback loop between TAMs and tumor cells. Tumor exosomal cSERPINE2 was shuttled to TAMs and notably elevated MALT1 level, and then enhanced the secretion of IL-6 through activating NF-$\upkappa$B pathway, leading to increasing proliferation and invasion of breast cancer cells. Moreover, IL-6 in turn increased the EIF4A3 and CCL2 level within tumor cells in a positive feedback manner, further enhancing tumor cSERPINE2 biogenesis and promoting the recruitment of TAMs. More importantly, the PLGA-based si-cSERPINE2 nanoparticles effectively attenuate breast cancer progression in vivo.

## Supplementary Information

**Additional file 1: Fig. S1. **Analysis of the expression and character of cSERPINE2 in breast cancercells. **Fig. S2. **cSERPINE2 reshaped the immune microenvironment of breast cancer. **Fig. S3 **Tumor exosomal cSERPINE2 targeted macrophages to promote theproliferation and invasion of breast cancer cells. **Fig. S4.** Tumor exosomal cSERPINE2 upregulated the MALT1 expressionin macrophages by sponging miR‑513a-5p. **Fig. S5. **Tumor exosomal cSERPINE2 promoted the secretion of IL-6 in TAMs via activating the NF-κB pathway to promote the progression of breast cancer. **Fig. S6. **IL-6 secreted by TAMs promoted the expression of CCL2 in breastcancer cells via activating the JAK2-STAT3 pathway. **Fig. S7. **Characteristics of si-cSERPINE2-NPs and therapeutic efficacy of si-cSERPINE2-NPs in breast cancer invitro. **Fig. S8. **Toxicity evaluation of systemic injection of si-cSERPINE2-NPs in breast cancer. **Supplementary Table S1. **Correlation between cSERPINE2 expression and clinicopathological features in 136 patients withbreast cancer.** Supplementary Table S2. **Univariate and multivariate analyses of OS in breast cancer (*n*=136).** Supplementary Table S3. **Univariate and multivariate analyses of RFS in breast cancer (*n*=136).** Supplementary Table S4. **The primers (5’-3’) used for RT-qPCR in this study.** Supplementary Table S5. **The probe sequences (5’-3’) for FISH.** Supplementary Table S6. **Primers for EIF4A3 RIP.** Supplementary Table S7. **Primers for CHIP.** Supplementary Table S8. **The probe sequences (5’-3’) for RNA pull-down assay.** Supplementary Table S9. **Primers for in vitro transcription.** Supplementary Table S10. **Effective sequences (5’-3’) of lentivirus plasmids-overexpression.** Supplementary Table S11. **Effective sequences (5’-3’) of lentivirus plasmids-knockdown.** Supplementary Table S12. **miRNA mimics and inhibitor sequences (5’-3’).

## Data Availability

The data sets used and/or analyzed during the current study are available from the corresponding author on reasonable request.

## Abbreviations

circRNAs: Circular RNAs
TME: Tumor microenvironment
TAMs: Tumor associated macrophages
SERPINE2: Serpin family E member 2
qRT-PCR: Quantitative reverse transcription PCR
OS: Overall survival
RFS: Recurrence-free survival
FISH: Fluorescent in situ hybridization
RBPs: RNA-binding proteins
RIP: RNA immunoprecipitation
IHC: Immunohistochemistry
Csf1: Colony stimulating factor 1
CM: Conditioned media
BMDMs: Bone marrow derived macrophages
TEM: Transmission electron microscopy
DEGs: Differentially expressed genes
JAKs: Janus kinases
GSEA: Gene set enrichment analysis
PLGA: Poly lactic-co-glycolic acid
DLS: Dynamic light scattering
EMT: Epithelial-to-mesenchymal transition
TNBC: Triple-negative breast cancer
GPCRs: G protein-coupled receptors
HCC: Hepatocellular carcinoma
IL-6: Interleukin-6
EPR: Enhanced permeability and retention
ISH: In situ hybridization
H&E: Hematoxylin–eosin
IF: Immunofluorescence
ALT: Alanine transaminase
AST: Aspartate transaminase
Cr: Creatinine
BUN: Blood urea nitrogen
