# Supplementary material for: Targeting tumor exosomal circular RNA cSERPINE2 suppresses breast cancer progression by modulating MALT1-NF-𝜅B-IL-6 axis of tumor-associated macrophages
Source: J Exp Clin Cancer Res. 2023 Feb 17;42:48. doi: 10.1186/s13046-023-02620-5 (PMC9936722; doi:10.1186/s13046-023-02620-5)
Supplement: Supplementary file 1 — Additional file 1: Fig. S1. Analysis of the expression and character of cSERPINE2 in breast cancercells. Fig. S2. cSERPINE2 reshaped the immune microenvironment of breast cancer. Fig. S3 Tumor exosomal cSERPINE2 targeted macrophages to promote theproliferation and invasion of breast cancer cells. Fig. S4. Tumor exosomal cSERPINE2 upregulated the MALT1 expressionin macrophages by sponging miR‑513a-5p. Fig. S5. Tumor exosomal cSERPINE2 promoted the secretion of IL-6 in TAMs via activating the NF-κB pathway to promote the progression of breast cancer. Fig. S6. IL-6 secreted by TAMs promoted the expression of CCL2 in breastcancer cells via activating the JAK2-STAT3 pathway. Fig. S7. Characteristics of si-cSERPINE2-NPs and therapeutic efficacy of si-cSERPINE2-NPs in breast cancer invitro. Fig. S8. Toxicity evaluation of systemic injection of si-cSERPINE2-NPs in breast cancer. Supplementary Table S1. Correlation between cSERPINE2 expression and clinicopathological features in 136 patients withbreast cancer. Supplementary Table S2. Univariate and multivariate analyses of OS in breast cancer (n=136). Supplementary Table S3. Univariate and multivariate analyses of RFS in breast cancer (n=136). Supplementary Table S4. The primers (5’-3’) used for RT-qPCR in this study. Supplementary Table S5. The probe sequences (5’-3’) for FISH. Supplementary Table S6. Primers for EIF4A3 RIP. Supplementary Table S7. Primers for CHIP. Supplementary Table S8. The probe sequences (5’-3’) for RNA pull-down assay. Supplementary Table S9. Primers for in vitro transcription. Supplementary Table S10. Effective sequences (5’-3’) of lentivirus plasmids-overexpression. Supplementary Table S11. Effective sequences (5’-3’) of lentivirus plasmids-knockdown. Supplementary Table S12. miRNA mimics and inhibitor sequences (5’-3’). [file 13046_2023_2620_MOESM1_ESM.zip › 13046_2023_2620_MOESM1_ESM/Supplementary Materials-230214_ESM.docx]

**Supplementary Materials for**

**Targeting tumor exosomal circular RNA cSERPINE2 suppresses breast cancer progression by modulating MALT1-NF-κB-IL-6 axis of tumor-associated macrophages**

Boxuan Zhou^1,2,*^, Zhaohong Mo^3,*^, Guie Lai^1,*^, Xiaohong Chen^4^, Ruixi Li^5^, Runxin Wu^6^, Jia Zhu^7,🖂^, Fang Zheng^2,🖂^

**^🖂^ Correspondence:**

Fang Zheng

[zhengf9@mail.sysu.edu.cn](mailto:zhengf9@mail.sysu.edu.cn)

Jia Zhu

Zhujia888618@163.com

These authors contributed equally: Boxuan Zhou, Zhaohong Mo, Guie Lai

This PDF file includes:

Supplementary Materials and Methods

References

Supplementary Figures and Figure Legends

Supplementary Table S1 to S12

**MATERIALS AND METHODS**

Cell culture and treatment

The human breast cancer cell lines MDA-MB-231, MDA-MB-468, BT-474, BT-549, SK-BR-3 and MCF-7, the human mammary epithelial cell line MCF10A and the human monocytic cell line THP-1 were purchased from ATCC. The mouse breast cancer cell line EO771 was purchased from CH3 Biosystems.

For THP-1 differentiation, THP-1 cells were seeded in 6-well plates at a density of 2×10^5^ cells/mL and treated with 5 ng/mL PMA for 24 hours to polarize macrophages. Subsequently, PMA-THP-1 macrophages were cultured by the addition of conditioned medium or exosomes from breast cancer cells for another 24 hours (1).

For generation of mouse bone marrow-derived macrophages (BMDMs), bone marrow cells were collected from femurs and tibias of C57BL/6 mice as previously described (2). Primary mouse bone marrow cells were cultured in DMEM with 10% FCS and 1% penicillin/streptomycin and 20% L929 cell supernatant to differentiate bone marrow cells into BMDMs (3). BMDMs were treated with 100 ng/mL LPS (Cat# L4130, Sigma) or 20 ng/mL IL-4 (Cat#214-14, PeproTech) to alter polarization. For evaluation of the effect of EO771 cell conditioned medium, the medium for BMDMs was changed from bone marrow differentiation medium to EO771 conditioned medium (a mixture of 50% EO771 conditioned medium and 50% bone conditioned medium) for 5 days after the bone marrow cells were seeded.

Human and mouse tumor-associated macrophages and corresponding breast cancer cells cocultivation was conducted using a noncontact coculture transwell system for 24 hours, and TAMs and breast cancer cells were harvested for further analyses (4).

RNA isolation, quantitative real-time PCR (RT-qPCR) and nucleic acid electrophoresis

Total RNA was isolated from tissues and cell lines with TRIzol reagent (Invitrogen, USA) according to the supplier’s protocol. Reverse transcription was performed using the PrimeScript RT master mix kit (TaKaRa, Japan) with random primers. For miRNAs, reverse transcription was performed using PrimeScript RT Reagent Kit with specific stem-loop primers. Real-time qPCR was carried out as previously described (5). β-actin and U6 were utilized as endogenous controls. The 2^-ΔΔCT^ method was used to calculate the relative RNA expression. Related primers are listed in supplementary file Table S4**.**

RNase R treatment and actinomycin D assay

Total RNA was extracted and then treated with RNase R (Lucigen, Middleton, WI, USA) at 37°C for 15 minutes according to the manufacturer’s instructions. Then, RT-qPCR was performed to evaluate the RNase R resistance of cSERPINE2 (6).

Breast cancer cells were seeded in 6-well plates at a density of 2×10^5^ cells/well. Cells were treated with actinomycin D (1 μg/ml) and harvested after 0, 4, 8, 12 and 24 hours. The relative RNA expression of cSERPINE2 and SERPINE2 were analyzed by RT-qPCR and normalized to the values of the 0-hour group (6).

Fluorescence in situ hybridization (FISH) and in situ hybridization (ISH)

Breast cancer cells were seeded onto sterile glass slides for at least 24 hours, fixed in 4% paraformaldehyde for 15 minutes at room temperature and permeabilized with PBS containing 0.05% Triton X-100 for 5 minutes at 4℃. For paraffin-embedded sections, slides were dewaxed and rehydrated. Then, the cells or slides were incubated with prehybridization solution for 2 hours at 50℃ before hybridization. Next, the sections were hybridized with an anti-cSERPINE2 oligodeoxynucleotide probe conjugated with DIG for 16 hours at 50℃ in hybridization solution. After washes in 2×SSC for 5 minutes and 50% deionized formamide/4×SSC for 25 minutes, fluorescein-conjugated antibodies against DIG were incubated overnight at 4℃, and Hoechst 33342 was used to counterstain the nuclei. Finally, images were captured by laser scanning confocal microscopy (LSM800, Zeiss). For ISH, converter POD- conjugated antibodies against DIG were added and incubated overnight at 4℃. After thorough washes with PBS, the sections were incubated with hydrogen peroxide for 15 min. Subsequently, HRP-conjugated rabbit anti-sheep antibody was added for 1h at room temperature. Immunodetection was performed using DAB, and images were captured.

For calculation of ISH staining scores, the intensity and proportion of cSERPINE2-positive cells were recorded. The proportion of positively stained tumor cells in slides was grades as follows: 0, no positive cells; 1, <25%; 2, 25-50%; 3, 50-75%; 4, >75%. The cells at each staining intensity were recorded on a scale of 0 (no staining), 1 (light brown), 2 (brown) and 3 (dark brown). ISH staining scores =staining intensity × proportion of positively stained cells. The sequences of probes used in this study are listed in supplementary file Table S5.

Hematoxylin-eosin (H&E), immunofluorescence (IF) and immunohistochemistry (IHC)

For H&E staining, paraffin-embedded samples were cut into 4 μm slides and deparaffinized and rehydrated; subsequently, sections were stained with hematoxylin- eosin as previously described (7). In addition, antigens were retrieved through a pressure cooker for 3 minutes in 0.01 M citrate buffer (pH =6.0) or EDTA buffer (pH =8.0). Sections were blocked in PBS with 5% BSA for 25 minutes at room temperature and incubated with antibodies specific for EIF4A3 (1:200, Cat#A4338, ABclonal), CD8 (1:50, Cat#A0663, ABclonal), CD19 (1:5000, Cat#A19013, ABclonal), CD56 (1:100, Cat#A7913, ABclonal), CD68 (1:50, Cat#A13286, ABclonal), Ki67 (1:200, Cat#12202, CST), CD44 (1:200, Cat#37259, CST), P65 (1:800, Cat#3033, CST), MALT1 (1:50, Cat#A2144, ABclonal) , and F4/80 (1:200, Cat#70076, CST) overnight at 4℃. Then, Alexa Fluor-conjugated or HRP-conjugated secondary antibodies were added and incubated at room temperature for 1h. The nucleus was stained with Hoechst 33342. Finally, images were captured by laser scanning confocal microscopy (LSM800, Zeiss). For immunohistochemistry, immunodetection was conducted using the DAB method.

RNA immunoprecipitation (RIP) assay

RIP was carried out using a Magna RIP RNA-Binding Protein Immunoprecipitation Kit (Cat#17-700, Millipore) as previously described (8). Briefly, 2×10^7^ cells were washed in ice-cold PBS, lysed with IP lysis buffer containing protease-inhibited cocktail and RNase inhibitor, and then incubated with 5 μg of rabbit polyclonal anti-EIF4A3 (Cat#17504-1-AP, Proteintech) or rabbit IgG control polyclonal antibody (Cat#30000-o-AP, Proteintech) overnight at 4℃. Then, the coprecipitated immunocomplexes were pulled down by protein A/G magnetic beads (Cat#88802, Thermo Fisher Scientific), and the enriched RNAs were subjected to RT-qPCR analysis. Related primers are listed in supplementary file Table S6.

ChIP assay

A ChIP Assay Kit (Cat#26157, Themo Fisher Scientific) was utilized to perform ChIP assays as previously described (9). Briefly, cells were seeded in 100 mm cell culture plates to 80% confluence and fixed in 1% formaldehyde at room temperature for 10 minutes. The fixed cells were harvested, lysed and sonicated for 10 cycles of 30s on/45s off using an Omni Ruptor 250 Ultrasonic Homogenizer (Omni International, Marietta, USA). The lysates were incubated with primary antibody against STAT3 (Cat#12640S, CST) or negative control rabbit IgG (Cat#3900, CST) overnight at 4℃. Protein A/G magnetic beads (Cat#88802, Thermo Fisher Scientific) were added to the reactions to bind the protein-conjugated antibody. The immunoprecipitated complex was eluted from magnetic beads, and the DNA was extracted by the proteinase K-chloroform method and subjected to PCR amplification. The primer sequences used in the ChIP assay are described in Table S7.

RNA pull-down assay

RNA pull-down assays were conducted as previously described (10). cSERPINE2 was *in vitro* transcribed by conjugation with biotin labeling using T7 RNA polymerase (Promega, Madison, Wisconsin, USA). Cell lysates were incubated with biotinylated cSERPINE2 oligo probes overnight at 4℃. For the miR-153a-5p pull-down assay, cells were transfected with biotin-labeled miRNA mimics or control (Generay, China) for 24 hours, and the cells were harvested and sonicated. Next, streptavidin magnetic beads were added to each binding reaction and further incubated for 1 hour at 37℃. The beads were thoroughly washed with wash buffer for five times. The coprecipitated RNA was extracted by the proteinase K-chloroform method and subjected to RT-qPCR analysis.

For the *in vitro* RNA/protein interaction assay, pre-mRNA of SERPINE2 was truncated into different versions, which were cloned into the pUC57-Amp vector with the T7 promoter. The truncated RNA sequence was *in vitro* transcribed by conjugation with biotin labeling using T7 RNA polymerase (Promega, Madison, Wisconsin, USA) following the manufacturer’s protocol. Next, five picomoles of 3’-biotinylated transcribed RNA was incubated with streptavidin magnetic beads and then interacted with 1μg recombinant EIF4A3 protein (Cat#Ag11130, Proteintech). The retrieved protein was detected by western blotting, and biotinylated RNA input was detected in northern blots using a Chemiluminescent Nucleic Acid Detection Module Kit (Cat#89880, Thermo Fisher Scientific). The detailed sequences of DNA oligo probes and *in vitro* expression vectors are listed in Table S8 and S9.

Western blotting

The protein lysates were extracted from the whole cells using RIPA lysis buffer, resolved on 4-12% SDS-polyacrylamide gels and then transferred to polyvinylidene difluoride membranes (Millipore, Billerica, MA, USA). Primary antibodies specific for EIF4A3 (1:10000, Cat#67740-1-Ig, Proteintech), CD63 (1:200; sc-5275, Santa Cruz), CD81 (1:1000, Cat#A5270, ABclonal), MALT1 (1:1000, Cat#A2144, ABclonal), p-IKKβ (1:1000, Cat#ab194519, Abcam), IKK-β (1:1000, Cat#8943, CST), p-IKBα (1:1000, Cat#2859, CST), IKBα (1:1000, Cat#4814, CST), p-JAK2 (1:1000, Cat#AP0373, ABclonal) , JAK2 (1:1000, Cat# A19629, ABclonal), p-STAT3 (1:1000, Cat#AP0070, ABclonal), STAT3 (1:1000, Cat#A1192, ABclonal) and GAPDH (1:1000, Cat#AC001, ABclonal) were used. Peroxidase-conjugated secondary antibody was used, and the antigen-antibody reaction was visualized by enhanced chemiluminescence assay (ECL, Thermo Fisher Scientific). For densitometry analysis including MALT1, IKK-β, IκBα, p−IκBα, JAK2 and STAT3, GAPAD was used as loading control. For protein quantification of p-IKK-β, p-JAK2 and p-STAT3, the relative level of phosphorylated fraction to total fraction was calculated.

Transwell invasion assay and EdU assay

The tumor cell invasion assay was performed with transwell migration chambers (pore size 8μm, diameter 6.5mmm polycarbonate membrane, Corning, USA), and a 50 μl dilution of Matrigel (Matrigel: serum-free DMEM 1:3) was precoated in the transwell membranes and solidified at 37℃ for 30 minutes. Cells suspended in 100μl serum-free medium were added to the upper chamber. In the lower chamber, 600 μl medium containing 10% FBS was added. After 20 hours of incubation in 5% CO2 at 37℃, the cells in the upper chamber were removed with a cotton swab. The cells on the lower side of the membrane were fixed with 4% formaldehyde and stained with 0.5% crystal violet. The stained cells were counted under a microscope in five randomly selected fields.

For the EdU assay, cells were seeded in 96-well plates at the desired density. Then, 10 μM EdU was added to the cells for 2 hours at 37℃ before fixation and permeabilization, and EdU staining was performed as previously described (11). The cell nuclei were stained with Hoechst 33342 for 15 minutes. Finally, images were captured by fluorescence microscopy.

ELISA assay

For validation of the IL-6 concentration, IL-6 levels in conditioned media were analyzed using commercially available ELISA kits (Cat#E-EL-H6156, Elabscience) according to the manufacturer’s instructions. In brief, cells-conditioned media was collected and centrifuged at 1000 × g for 20 minutes to remove cell debris, and then, 200μl of supernatant was used in the ELISA.

Luciferase reporter assay

A luciferase reporter assay was performed using a dual-luciferase reporter vector containing Renilla luciferase (hRluc) and firefly luciferase gene (hLuc+) as previously described (12, 13). The 3’ UTR sequences of MALT1 and cSERPINE2 were cloned into the hRluc cassette downstream. For the EIF4A3 promoter assay, a 2000-bp DNA fragment containing STAT3 binding sites upstream from the EIF4A3 promoter was cloned into the hRluc cassette downstream. Mutations were generated in the binding sites. The miRNA mimics and inhibitor were obtained from Generay (Shanghai, China). The luciferase reporter plasmids were cotransfected into the indicated cells with the indicated treatment. Finally, the luciferase activities were quantified with a dual-luciferase reporter assay (Promega).

Plasmid construction, stable cell line generation and oligonucleotide transfection

For construction of shcSERPINE2, si-cSERPINE2 was cloned into the pLKO.1 vector. For generation of the EIF4A3 overexpression vector, the full-length ORF sequence was subcloned into the pcDNA 3.1(+) vector. For cSERPINE2 overexpression, the cDNA of cSERPINE2 was cloned into pLO-ciR vector. For generation of stable cell lines, lentiviruses containing the above vectors were generated in HEK293T cells and cancer cells transduction was performed as previously described (6). si-DHX9, si-QKI, si-ADAR-1, si-EIF4A3, si-FUS, si-HnRNPM, si-STAT3, miR-513a-5p inhibitors, miR-513a-5p mimics and their negative controls were designed and synthesized by Generay Biotech (Shanghai, China). The above miRNAs and siRNA transfection was performed by LipofectamineTM RNAiMAX Reagent (Invitrogen, CA, USA) according to the manufacturer’s recommendation. All sequences used in this study are summarized in Supplementary Table S10 – S12.

Library construction for RNA sequencing and sequencing procedure

RNA sequencing and bioinformatics analysis were performed by Genergy Biotechnology Co., Ltd. (Shanghai, China). Briefly, total RNA was isolated from TAMs and tumor cells with the indicated treatments using TRIzol reagent. RNA was amplified and transcribed to create the final cDNA library. Next-generation sequencing of cDNA libraries was performed on the Illumina NovaSeq 6000 platform.

Preparation and characterization of NPs

The lipid-polymer hybrid NPs loaded with si-cSERPINE2 were prepared by an innovative and robust self-assembly strategy (14-16). In brief, 5 mg PLGA and 0.5 mg lipid-like compound G0-C14 were dissolved in 1 mL of dimethylformamide (DMF). Subsequently, si-cSERPINE2 was mixed with the organic solution to form siRNA/G0-C14 nanocomplexes. Then, the polymer solution with the siRNA/G0-C14 nanocomplexes was added to a 20 mL aqueous solution containing 2 mg DSPE-PEG. The above suspension was thoroughly stirred at 1000 rpm for 30 minutes, and the mixture was added dropwise to 5 mL of deionized water. For removal of the remaining organic solvent and free compounds, the hybrid NPs dispersion was transferred to an ultrafiltration device (EMD Millipore, MWCO 100kDa) and centrifuged. After washes with ice-cold deionized water, the obtained NPs were collected and dispersed in PBS solution. The particle size and zeta potential were examined by dynamic light scattering. The NPs morphology was viewed on a transmission electron microscope.

Antitumor efficacy and toxicity evaluation of si-cSERPINE2 NPs *in vivo*

For investigation of the antitumor efficacy of the si-cSERPINE2 loaded NPs *in vivo*, breast cancer orthotopic models were created as described above. When the tumors reached ~120mm^3^ (about day 10), si-cSERPINE2 or si-ctrl NPs (700 μg per kg of mouse weight) were injected into the mice through the tail vein every three days for six cycles (14). For further investigation of the suppressive effect of the si-cSERPINE2 loaded NPs on tumor metastasis *in vivo*, breast cancer lung metastasis models were established. After 3 weeks of tail vein injection of EO771 cells, si-cSERPINE2 or si-ctrl NPs were injected into the mice through the tail vein every three days for six cycles.

For evaluation of the toxicity of si-cSERPINE2 NPs *in vivo*, we harvested the major organs, including the liver, kidney, lung, spleen and heart, and further performed hematoxylin-eosin (H&E) staining. Additionally, the levels of mouse blood alanine transaminase (ALT), aspartate transaminase (AST), creatinine (Cr) and blood urea nitrogen (BUN) were analyzed to test the hepatic and renal toxicity of si-cSERPINE2 NPs.

Quantification of lung metastases

After being inflated using 4% paraformaldehyde, lungs were embedded in paraffin upon tissue fixation in 4% paraformaldehyde. The metastatic burden was assessed by serial sectioning of the entire lungs. Hematoxylin and eosin staining was performed on sections. Images were acquired using AxioScan Z1 (Zeiss) slide scanner and analyzed using Zen Blue software.

Statistical analysis

All statistical analyses were carried out by applying SPSS (version 23.0, SPSS, Inc., Chicago, IL, USA) or GraphPad Prism (version 8.0, La Jolla, CA, USA). All data, unless otherwise noted, are presented as the mean ± standard deviation (SD). Comparisons were performed using Student’s t test, one-way ANOVA, or the Mann-Whitney U-test, as appropriate. Correlations between two groups were measured by Pearson correlation analysis. Survival curves were plotted using the Kaplan-Meier method and tested by the log-rank test. A Cox proportional hazards regression model was utilized to evaluate the potential independent prognostic factors. A two-tailed P value < 0.05 was considered statistically significant.

**Reference**

1. Cassetta L, Fragkogianni S, Sims AH, Swierczak A, Forrester LM, Zhang H, et al. Human Tumor-Associated Macrophage and Monocyte Transcriptional Landscapes Reveal Cancer-Specific Reprogramming, Biomarkers, and Therapeutic Targets. Cancer Cell. 2019;35(4):588-+.

2. Im JH, Buzzelli JN, Jones K, Franchini F, Gordon-Weeks A, Markelc B, et al. FGF2 alters macrophage polarization, tumour immunity and growth and can be targeted during radiotherapy. Nature Communications. 2020;11(1).

3. Cho D-I, Kim MR, Jeong H-y, Jeong HC, Jeong MH, Yoon SH, et al. Mesenchymal stem cells reciprocally regulate the M1/M2 balance in mouse bone marrow-derived macrophages. Experimental and Molecular Medicine. 2014;46.

4. Wei C, Yang C, Wang S, Shi D, Zhang C, Lin X, et al. Crosstalk between cancer cells and tumor associated macrophages is required for mesenchymal circulating tumor cell-mediated colorectal cancer metastasis. Molecular cancer. 2019;18.

5. Liu B, Sun L, Liu Q, Gong C, Yao Y, Lv X, et al. A cytoplasmic NF-κB interacting long noncoding RNA blocks IκB phosphorylation and suppresses breast cancer metastasis. Cancer Cell. 2015;27(3):370-81.

6. Chen RX, Chen X, Xia LP, Zhang JX, Pan ZZ, Ma XD, et al. N(6)-methyladenosine modification of circNSUN2 facilitates cytoplasmic export and stabilizes HMGA2 to promote colorectal liver metastasis. Nat Commun. 2019;10(1):4695.

7. Fischer AH, Jacobson KA, Rose J, Zeller R. Hematoxylin and eosin staining of tissue and cell sections. CSH protocols. 2008;2008:pdb.prot4986.

8. Jin X, Xu XE, Jiang YZ, Liu YR, Sun W, Guo YJ, et al. The endogenous retrovirus-derived long noncoding RNA TROJAN promotes triple-negative breast cancer progression via ZMYND8 degradation. Science advances. 2019;5(3):eaat9820.

9. Xiang DM, Sun W, Ning BF, Zhou TF, Li XF, Zhong W, et al. The HLF/IL-6/STAT3 feedforward circuit drives hepatic stellate cell activation to promote liver fibrosis. Gut. 2018;67(9):1704-15.

10. Zhang PF, Gao C, Huang XY, Lu JC, Guo XJ, Shi GM, et al. Cancer cell-derived exosomal circUHRF1 induces natural killer cell exhaustion and may cause resistance to anti-PD1 therapy in hepatocellular carcinoma. Molecular cancer. 2020;19(1):110.

11. Xia X, He J, Liu B, Shao Z, Xu Q, Hu T, et al. Targeting ERα degradation by L-Tetrahydropalmatine provides a novel strategy for breast cancer treatment. International journal of biological sciences. 2020;16(12):2192-204.

12. Hong XY, Wan HL, Li T, Zhang BG, Li XG, Wang X, et al. STAT3 ameliorates cognitive deficits by positively regulating the expression of NMDARs in a mouse model of FTDP-17. Signal Transduction and Targeted Therapy. 2020;5(1).

13. Jiang Y, Zhou JP, Zhao JS, Zhang HY, Li L, Li H, et al. TheU2AF2/circRNA ARF1/miR-342-3p/ISL2feedback loop regulates angiogenesis in glioma stem cells. Journal of Experimental & Clinical Cancer Research. 2020;39(1).

14. Islam MA, Xu YJ, Tao W, Ubellacker JM, Lim M, Aum D, et al. Restoration of tumour-growth suppression in vivo via systemic nanoparticle-mediated delivery of PTEN mRNA. Nature Biomedical Engineering. 2018;2(11):850-64.

15. Zhu X, Tao W, Liu D, Wu J, Guo ZL, Ji XY, et al. Surface De-PEGylation Controls Nanoparticle-Mediated siRNA Delivery In Vitro and In Vivo. Theranostics. 2017;7(7):1990-2002.

16. Zhu X, Xu YJ, Solis LM, Tao W, Wang LZ, Behrens C, et al. Long-circulating siRNA nanoparticles for validating Prohibitin1-targeted non-small cell lung cancer treatment. Proceedings of the National Academy of Sciences of the United States of America. 2015;112(25):7779-84.

**SUPPLEMENTARY FIGURE LEGEND**

**Fig. S1** Analysis of the expression and character of cSERPINE2 in breast cancer cells. **a** The relative expression of cSERPINE2 in breast cancer cells and normal breast cell line. **b** The levels of cSERPINE2 and linear SERPINE2 after treating with RNase R. **c** The levels of cSERPINE2 and linear SERPINE2 in MCF-7 and MDA-MB-468 cells treated with actinomycin D. **d** qRT-PCR analysis of cell fractions to certificate the subcellular localization of cSERPINE2 in MCF-7 and MDA-MB-468 cells. β-actin and U6 were applied as positive controls in the cytoplasm and nucleus, respectively. **e** The levels of RBPs in MDA-MB-468 cells after knocking down RBPs. **f, g** Representative images **(f)** and quantification **(g)** of western blotting for the expression of EIF4A3 in MDA-MB-468 cells as indicated treatments. **h** qRT-PCR was performed to detected the expression of cSERPINE2 in MDA-MB-468 cells as indicated treatments. **i** TCGA analysis of the EIF4A3 expression in breast cancer and control normal samples. **j** Kaplan-Meier analysis of the OS and RFS of breast cancer patients according to EIF4A3 expression based on Kaplan-Meier plot database. Data are presented as the means ± SD of three independent experiments. **P*<0.05, ***P* < 0.01, ****P* < 0.001.

**Fig. S2** cSERPINE2 reshaped the immune microenvironment of breast cancer. **a** The relative expression of cSERPINE2 and SERPINE2 in MDA-MB-468 and MCF-7 cells as indicated treatments. **b, c** The proliferation and invasion of MDA-MB-468 and MCF-7 cells as indicated treatments were evaluated using EdU assays and transwell invasion assays, respectively. Scale bar, 20 μm. **d** Sequence alignment identification of human and mouse cSERPINE2. **e** The relative expression of cSERPINE2 and SERPINE2 in EO771 cells as indicated treatments. **f, g** The proliferation and invasion of EO771 cells as indicated treatments were evaluated using EdU assays and transwell invasion assays, respectively. Data are presented as the means ± SD of three independent experiments. **P*<0.05, ***P* < 0.01, ****P* < 0.001.

**Fig. S3** Tumor exosomal cSERPINE2 targeted macrophages to promote the proliferation and invasion of breast cancer cells. **a, b** CM of MCF-7 and EO771 cells transduced by empty vector or cSERPINE2 overexpression vector was collected. PMA-THP-1 and BMDMs cells were induced into corresponding TAMs by these CM. qRT-PCR analysis of the expression of CD163, CD206, TNF-α, IL-1β and IL-6 in PMA-THP-1, BMDMs and their derived TAMs. **c, d** MCF-7 and EO771 cells were co-cultured with indicated TAMs for 24 h, and the proliferation and invasion of MCF-7 and EO771 cells were evaluated using EdU assays and transwell invasion assays, respectively. Data are presented as the means ± SD of three independent experiments. **P*<0.05, ***P* < 0.01, ****P* < 0.001.

**Fig. S4** Tumor exosomal cSERPINE2 upregulated the MALT1 expression in macrophages by sponging miR‑513a-5p. **a** The luciferase activity of cSERPINE2-WT or cSERPINE2-MUT in 293T cells after co-transfection with miR‑513a-5p mimic. **b** The percentage of TAMs with at least two events of co-localization of cSERPINE2 and miR-513a-5p. **c** Clustered heatmap of the top 200 up-regulated and down-regulated genes in TAM^OE-Exo^ compared to TAM^EV-Exo^ cells. **d** The luciferase activity of MALT1-WT or MALT1-MUT in 293T cells after co-transfection with miR‑513a-5p mimic. **e** MALT1 expression in multiple cancer types in the Human Protein Atlas database. **f** Respective IHC images showing the MALT1 expression on breast cancer tissues with low or high cSERPINE2 expression. Scale bar, 20 μm. **g, h** Western blotting analysis (left) and quantification (right) of MALT1 expression in PMA-THP-1 **(g)**, MCF-7 **(g)**, BMDMs **(h)** and EO771**(h)** cells with or without cSERPINE2 overexpression. Data are presented as the means ± SD of three independent experiments. **P*<0.05, ***P* < 0.01, ****P* < 0.001.

**Fig. S5** Tumor exosomal cSERPINE2 promoted the secretion of IL-6 in TAMs via activating the NF-κB pathway to promote the progression of breast cancer. **a** ELISA assays of IL-6 level in the supernatants from TAMs as indicated treatments. **b, c** Representative Western blotting images **(b)** and quantification **(c)** of MALT1, phosphorylated IKK, IKK-β, phosphorylated Iκ-Bα and Iκ-Bα levels in PMA-THP-1 and TAMs as indicated treatments. **d** Quantification the expression of MALT1, phosphorylated IKK, IKK-β, phosphorylated Iκ-Bα and Iκ-Bα through western blotting in BMDM and mTAMs as indicated treatments. **e** Orthotopic breast cancer models were injected exosomes derived from EO771 cells transduced by empty vector or cSERPINE2 overexpression vector, followed by treatment of IgG or anti-IL-6 antibody (n=6 for each group). The Ki67 and CD44 expression and F4/80^+^ macrophages infiltration in the tumor tissues were evaluated using IHC staining. **f, g** The representative Western blotting images **(f)** and quantification **(g)** of phosphorylated JAK2, JAK2, phosphorylated STAT3 ,STAT3 levels in orthotopic tumor tissues. **h, i** EO771 cells were co-cultured with indicated mTAMs, followed by treatment of IgG or anti-IL-6 antibody. Representative Western blotting images (h) and quantification (i) of phosphorylated JAK2, JAK2, phosphorylated STAT3, STAT3 levels in the EO771 cells. Data are presented as the means ± SD of three independent experiments. **P*<0.05, ***P* < 0.01, ****P* < 0.001.

**Fig. S6** IL-6 secreted by TAMs promoted the expression of CCL2 in breast cancer cells via activating the JAK2-STAT3 pathway. **a** The relative expression of CCL2 in MCF-7 cells cocultured with TAM^EV-Exo^ or cocultured with TAM^OE-Exo^. **b, c** The levels of EIF4A3 **(b)** and CCL2 (**c**) expression in MCF-7 cells as indicated treatments. **d** Representative Western blotting images (left) and quantification (right) of phosphorylated JAK2, JAK2, phosphorylated STAT3, STAT3 levels in MCF-7 cells as indicated treatments. **e** The relative expression of STAT3 in MCF-7 cells as indicated treatments. **f** MCF-7 cells were treated with IL-6 after STAT3 knockdown. The expression of CCL2 in MCF-7 cells was determined by qRT-PCR. **g** Sequence motif representing the consensus STAT3 binding motif (JASPAR database, upper) and schematic diagram of the putative STAT3 binding site in the CCL2 promoter (lower). **h** ChIP-qPCR was performed in MCF-7 cells cocultured with TAM^EV-Exo^ or cocultured with TAM^OE-Exo^ to detect the enrichment of potential binding sequences using the STAT3 antibody. **i, j** Schema for representing the experiment procedures (**i**) and transwell migration assays (**j**) showing the chemotaxis ability of PMA-THP-1 exposed to CM from MCF-7 cells cocultured with TAM^EV-Exo^ or cocultured with TAM^OE-Exo^, Scale bar, 20 μm. Data are presented as the means ± SD of three independent experiments. **P*<0.05, ***P* < 0.01, ****P* < 0.001.

**Fig. S7** Characteristics of si-cSERPINE2-NPs and therapeutic efficacy of si-cSERPINE2-NPs in breast cancer *in vitro.* **a** The cSERPINE2 expression determined by qRT-PCR in MDA-MB-468 cells treated with the PBS, NPs, si-ctrl-NPs, si-cSERPINE2-NPs or shcSERPINE2-1. **b** THP-1 cells were treated with PMA, followed by the treatment of exosomes derived from tumor cells as indicated treatments to obtain TAMs. Then tumor cells cocultured with these TAMs were used to subsequent experiments. **c, d** PMA-THP-1 cells were stimulated with exosomes from MDA-MBA-468 cells as indicated treatments to obtain TAMs. The cSERPINE2 (**c**) and MALT1 (**d**) expression in TAMs were determined by qRT-PCR. **e, f** EO771 cells were cocultured with indicated mTAMs for 24 h, and proliferative and invasive ability of EO771 cells were evaluated using EdU assays **(e)** and transwell invasion assays **(f)**, respectively. **g, h** MDA-MB-468 cells were cocultured with indicated TAMs for 24 h, and proliferative and invasive ability of MDA-MB-468 were evaluated using EdU assays **(g)** and transwell invasion assays **(h)**, respectively. **i** Circulation profile of naked Cy5-si-cSERPINE2-1 and Cy5-si-cSERPINE2-1 NPs after i.v. administration. **j** Biodistribution of different Cy5-siRNA injections on major organs and tumors after 24 h of treatment. Data are presented as the means ± SD of three independent experiments. **P*<0.05, ***P* < 0.01, ****P* < 0.001.

**Fig. S8** Toxicity evaluation of systemic injection of si-cSERPINE2-NPs in breast cancer. **a** The expression of cSERPINE2 in orthotopic tumor tissues as indicated treatments was determined by qRT-PCR. **b** Body weight changes of orthotopic breast cancer models (left) and lung metastasis of breast cancer models (right) during experimental period. **c** Blood ALT, AST, BUN and Cr in the orthotopic breast cancer models after six consecutive injections of PBS, NPs, si-ctrl NPs or si-cSERPINE2-1 NPs. Data are presented as the means ± SD of three independent experiments. **P*<0.05, ***P* < 0.01, ****P* < 0.001.

**Supplementary Table S1.**

Correlation between cSERPINE2 expression and clinicopathological features in 136 patients with breast cancer.

| Variables | cSERPINE2 | | Chi-square | *P-*value |
| --- | --- | --- | --- | --- |
|  | Low（68） | High（68） |  |  |
| Age |  |  | 0.763 | 0.382 |
| ≤ 45 | 25 | 30 |  |  |
| > 45 | 43 | 38 |  |  |
| Menstruation status |  |  | 0.477 | 0.490 |
| Premenopause | 36 | 40 |  |  |
| Menopause | 32 | 28 |  |  |
| Grade |  |  | 4.841 | **0.028** |
| I-II | 57 | 46 |  |  |
| III | 11 | 22 |  |  |
| Tumor size |  |  | 8.935 | **0.003** |
| ≤ 2cm | 50 | 33 |  |  |
| > 2cm | 18 | 35 |  |  |
| Lymph node |  |  | 5.225 | **0.022** |
| Negative | 48 | 35 |  |  |
| Positive | 20 | 33 |  |  |
| TNM stage |  |  | 5.440 | **0.020** |
| I-II | 56 | 44 |  |  |
| III | 12 | 24 |  |  |

**Supplementary Table S2.**

Univariate and multivariate analyses of OS in breast cancer (n=136).

|  | Univariate | Multivariate | | |
| --- | --- | --- | --- | --- |
|  | *P* | HR | 95%CI | *P* |
| Age, year (> 45) | 0.418 |  |  |  |
| Premenopause | 0.598 |  |  |  |
| Grade (III) | 0.250 |  |  |  |
| Tumor size (> 2cm) | **<0.001** | 2.457 | 1.017-5.932 | **0.046** |
| Lymph node (positive) | **<0.001** | 3.258 | 1.388-7.646 | **0.007** |
| TNM stage (III) | **0.016** | 0.789 | 0.337-1.851 | 0.586 |
| cSERPINE2 (high) | **0.001** | 2.643 | 1.127-6.199 | **0.025** |

**Supplementary Table S3.**

Univariate and multivariate analyses of RFS in breast cancer (n=136).

|  | Univariate | Multivariate | | |
| --- | --- | --- | --- | --- |
|  | *P* | HR | 95%CI | *P* |
| Age, year (> 45) | 0.508 |  |  |  |
| Premenopause | 0.683 |  |  |  |
| Grade (III) | **0.024** | 0.876 | 0.433-1.774 | 0.713 |
| Tumor size (> 2cm) | **<0.001** | 3.433 | 1.508-7.814 | **0.003** |
| Lymph node (positive) | **<0.001** | 2.684 | 1.263-5.705 | **0.010** |
| TNM stage (III) | **<0.001** | 1.649 | 0.801-3.397 | 0.175 |
| cSERPINE2 (high) | **<0.001** | 2.171 | 1.020-4.624 | **0.044** |

**Supplementary Table S4.**

The primers (5’-3’) used for RT-qPCR in this study.

| Primers for RT-qPCR | 5’-3’ | |
| --- | --- | --- |
| cSERPINE2(human) | Forward | AGCCTCTGCCTGTGATTCCATC |
|  | Reverse | GGGAGATGCCAGTTCATGGTT |
| cSERPINE2(mouse) | Forward | CTCTGCCTCTGAGTCCATCAA |
|  | Reverse | GCCAATTCATGGTTCCTTCCA |
| SERPINE2 | Forward | TTTGCAAAAATAACAACAGGGTCA |
|  | Reverse | GCTGCTGAAGCTTTGGTTCC |
| miR-513a-5p | Forward | AGGGAGGTGTCATCTCAACTGA |
|  | Reverse | CTCACAGTACGTTGGTATCCTTGTG |
| miR-361-3p | Forward | CGGGCTCCCCCAGGTGTGATT |
|  | Reverse | CAGCCACAAAAGAGCACAAT |
| miR-324-5p | Forward | GCCCGCTCTTTGGTTATCTAG |
|  | Reverse | CTCACAGTACGTTGGTATCCTTGTG |
| b-actin | Forward | GGGAAATCGTGCGTGACATTAAG |
|  | Reverse | TGTGTTGGCGTACAGGTCTTTG |
| U6 | Forward | GCTTCGGCAGCACATATACTAAAAT |
|  | Reverse | CGCTTCACGAATTTGCGTGTCAT |
| DHX9 | Forward | TCCAACTGGAATCCTTGGAC |
|  | Reverse | TTTTCCCACATCCAGTAGCC |
| QKI | Forward | GGGGAAATGGAAACGAAGG |
|  | Reverse | TTGAGCCTTTGCCTCGGAC |
| EIF4A3 | Forward | GGGCATCTACGCTTACGGTT |
|  | Reverse | GAGCAAGCAGCCCCTGAATA |
| FUS | Forward | CTATGGAACTCAGTCAACTCCCC |
|  | Reverse | CTGCCCGTAAGACGATTGG |
| ADAR-1 | Forward | CTGAGACCAAAAGAAACGCAGA |
|  | Reverse | GCCATTGTAATGAACAGGTGGTT |
| HnRNPM | Forward | GAGCCATATGCCAATCCAAC |
|  | Reverse | AGCGTCCATTAAGAGCTCCA |
| CD163(human) | Forward | TTTGTCAACTTGAGTCCCTTCAC |
|  | Reverse | TCCCGCTACACTTGTTTTCAC |
| *Cd163*(mouse) | Forward | GGTGGACACAGAATGGTTCTTC |
|  | Reverse | CCAGGAGCGTTAGTGACAGC |
| CD206(human) | Forward | GGGTTGCTATCACTCTCTATGC |
|  | Reverse | TTTCTTGTCTGTTGCCGTAGTT |
| *Cd206*(mouse) | Forward | CTCTGTTCAGCTATTGGACGC |
|  | Reverse | TGGCACTCCCAAACATAATTTGA |
| TNF-α(human) | Forward | TCTCGAACCCCGAGTGACAA |
|  | Reverse | TGAAGAGGACCTGGGAGTAG |
| *Tnf-α*(mouse) | Forward | CTGAACTTCGGGGTGATCGG |
|  | Reverse | GGCTTGTCACTCGAATTTTGAGA |
| IL-1β(human) | Forward | TGAACTGAAAGCTCTCCACC |
|  | Reverse | CTGATGTACCAGTTGGGGAA |
| *Il-1β*(mouse) | Forward | CCTTCATCTTTGAAGAAGAGCCC |
|  | Reverse | AATGGGAACGTCACACACCA |
| IL-6(human) | Forward | AATAACCACCCCTGACCCAAC |
|  | Reverse | ACATTTGCCGAAGAGCCCT |
| *Il-6*(mouse) | Forward | CTGCAAGAGACTTCCATCCAG |
|  | Reverse | AGTGGTATAGACAGGTCTGTTGG |
| MALT1(human) | Forward | TGGAAGCCCTATTCCTCACTACC |
|  | Reverse | CATGACACCAGTAGGTTCCTTGG |
| *Malt1*(mouse) | Forward | GACAGTCAAGATAGCAAGAAGGC |
|  | Reverse | TGGTCAATTCATACACATCCACC |
| CCL2 | Forward | AAGATCTCAGTGCAGAGGCTCG |
|  | Reverse | CACAGATCTCCTTGGCCACAA |
| STAT3 | Forward | ACCAGCAGTATAGCCGCTTC |
|  | Reverse | GCCACAATCCGGGCAATCT |
| DDX58 | Forward | AGACCCTGGACCCTACCTACA |
|  | Reverse | CTCCATTGGGCCCTTGTTGT |
| BCL2 | Forward | GAGGATTGTGGCCTTCTTTG |
|  | Reverse | AGGTACTCAGTCATCCACA |
| PRKCB | Forward | ATCGCCCCCGAGATAATTGC |
|  | Reverse | GGATGGCGGGTGAAAAATCG |
| NFKBIA | Forward | AAGTGGTCCGCCAAGTGAAG |
|  | Reverse | CGATTTCTGGCTGGTTAGTGATC |
| PLAU | Forward | GCTTGTCCAAGAGTGCATGGT |
|  | Reverse | CAGGGCTGGTTCTCGATGG |
| VCAM1 | Forward | GTCTCCAATCTGAGCAGCAA |
|  | Reverse | TGGGAAAAACAGAAAAGAGGTG |

**Supplementary Table S5.**

The probe sequences (5’-3’) for FISH.

| Probes | 5’-3’ |
| --- | --- |
| cSERPINE2 | DIG-AAAAATGAAACCAGGGGTCGTCCTTGGTGGAA-DIG |
| miR-513a-5p | Cy5-UUCACAGGGAGGUGUCAU-Cy5 |

**Supplementary Table S6.**

Primers for EIF4A3 RIP.

| Primers | 5’-3’ | |
| --- | --- | --- |
| cSERPINE2-a | Forward | GGACCTGGCACATTGTAAGGA |
|  | Reverse | TTTCAAAACACACCCTGGGGA |
| cSERPINE2-b | Forward | GGATTTGCTGGTGCCACTTG |
|  | Reverse | GATCACAGCGCAACTCAACC |
| cSERPINE2-c | Forward | GCCTCTTGCTTCTGCCATTG |
|  | Reverse | TTGAGATGTGGAAAGGGCTGT |
| cSERPINE2-d | Forward | TGGAGGTCCAGTTGGAAACA |
|  | Reverse | ACTCCCATCCTTCACATTCTCC |
| H19lncRNA | Forward | CTTCTGGGCTCAAGTGATCCT |
|  | Reverse | TTGTGCCATGAGACTCCATCAG |

**Supplementary Table S7.**

Primers for CHIP.

| Primers | 5’-3’ | |
| --- | --- | --- |
| EIF4A3-BS1 | Forward | TATTCGAGGCGGGGCTATCT |
|  | Reverse | CGTGTGGACCTGAATTCCCT |
| EIF4A3-BS2 | Forward | CTGGTTAAAGTTCTGCTGGGC |
|  | Reverse | TTGTCAGTAGACGGTGCTGC |
| CCL2-BS1 | Forward | AATGCATTGTCAGGGAGCCG |
|  | Reverse | CAGCTTACCTTCAGGCCACA |
| CCL2-BS2 | Forward | TGCTCATTTGGTCTCAGCAGT |
|  | Reverse | ACTTTCATGCTGGAGGCGAG |

**Supplementary Table S8.**

The probe sequences (5’-3’) for RNA pull-down assay.

| Probes for RNA pull-down | 5’-3’ |
| --- | --- |
| control-biotin (anti-sense） | Biotin-GACACCUCCCUGUGAAUU |
| miR-513a-5p-biotin (sense） | Biotin-UUCACAGGGAGGUGUCAU |
| control-biotin (anti-sense） | Biotin-TTCCACCAAGGACGACCCCTGGTTTCATTTTT |
| cSERPINE2-biotin (sense） | Biotin-AAAAATGAAACCAGGGGTCGTCCTTGGTGGAA |

**Supplementary Table S9.**

Primers for *in vitro* transcription.

| Primers | 5’-3’ | |
| --- | --- | --- |
| cSERPINE2-F1(-3850 to -3700) | Forward | TAATACGACTCACTATAGGGG  CATTTAAGTATAATTTAAATATTTTT |
|  | Reverse | AAAATCCTCGAGAGACAGAGGATTG  AAGTGGGAGCAGATGG |
| cSERPINE2-F2(-2850 to -2700) | Forward | TAATACGACTCACTATAGGGG  GCAAAGACTTGGAACCAACCCGAATG |
|  | Reverse | AAAAGAATGATGGTTTCCAGCTTCATC  CATGTCCCTGCAAG |
| cSERPINE2-F3(-50 to +100) | Forward | TAATACGACTCACTATAGGGG  AGCCTCTGCCTGTGATTCCATCAATG |
|  | Reverse | AAAAGCCATTGAATTGGACTTGCAGA  CACCACTTTGAACCC |
| cSERPINE2-F4(+350 to +500) | Forward | TAATACGACTCACTATAGGGG  TTACCTGGAATGTCCTCCTCATCCTT |
|  | Reverse | AAAAGGCTGTAATCGGGGGGGTGTA  AAAGTTGTAATCATGT |
| cSERPINE2-F5(+850 to +1000) | Forward | TAATACGACTCACTATAGGGG  CTGGGCAACAGAATGAGACTGTGTCT |
|  | Reverse | AAAATCCCATCCTTCACATTCTCCTGT  TGTTCCAGACCTAC |

**Supplementary Table S10.**

Effective sequences (5’-3’) of lentivirus plasmids-overexpression.

| Plasmids | 5’-3’ |
| --- | --- |
| LV-EIF4A3 | ACAGCGAGGTCGGCAGCGGCACAGCGAGGTCGGCAGCGGCACAGCGAGGTCGGCAGCGGCACAGCGAGGTCGGCAGCGGCAGCGAGGTCGGCAGCGGCACAGCGAGGTCGGCAGCGGCAGCGAGGTCGGCAGCGGCGCGCGCTGTGCTCTTCCGCGGACTCTGAATCATGGCGACCACGGCCACGATGGCGACCTCGGGCTCGGCGCGAAAGCGGCTGCTCAAAGAGGAAGACATGACTAAAGTGGAATTCGAGACCAGCGAGGAGGTGGATGTGACCCCCACGTTCGACACCATGGGCCTGCGGGAGGACCTGCTGCGGGGCATCTACGCTTACGGTTTTGAAAAACCATCAGCAATCCAGCAACGAGCAATCAAGCAGATCATCAAAGGGAGAGATGTCATCGCACAGTCTCAGTCCGGCACAGGAAAAACAGCCACCTTCAGTATCTCAGTCCTCCAGTGTTTGGATATTCAGGGGCTGCTTGCTCTCGGTGACTACATGAATGTCCAGTGCCATGCCTGCATTGGAGGCACCAATGTTGGCGAGGACATCAGGAAGCTGGATTACGGACAGCATGTTGTCGCGGGCACTCCAGGGCGTGTTTTTGATATGATTCGTCGCAGAAGCCTAAGGACACGTGCTATCAAAATGTTGGTTTTGGATGAAGCTGATGAAATGTTGAATAAAGGTTTCAAAGAGCAGATTTACGATGTATACAGGTACCTGCCTCCAGCCACACAGGTGGTTCTCATCAGTGCCACGCTGCCACACGAGATTCTGGAGATGACCAACAAGTTCATGACCGACCCAATCCGCATCTTGGTGAAACGTGATGAATTGACTCTGGAAGGCATCAAGCAATTTTTCGTGGCAGTGGAGAGGGAAGAGTGGAAATTTGACACTCTGTGTGACCTCTACGACACACTGACCATCACTCAGGCGGTCATCTTCTGCAACACCAAAAGAAAGGTGGACTGGCTGACGGAGAAAATGAGGGAAGCCAACTTCACTGTATCCTCAATGCATGGAGACATGCCCCAGAAAGAGCGGGAGTCCATCATGAAGGAGTTCCGGTCGGGCGCCAGCCGAGTGCTTATTTCTACAGATGTCTGGGCCAGGGGGTTGGATGTCCCTCAGGTGTCCCTCATCATTAACTATGATCTCCCTAATAACAGAGAATTGTACATACACAGAATTGGGAGATCAGGTCGATACGGCCGGAAGGGTGTGGCCATTAACTTTGTAAAGAATGACGACATCCGCATCCTCAGAGATATCGAGCAGTACTATTCCACTCAGATTGATGAGATGCCGATGAACGTTGCTGATCTTATCTGAAGCAGCAGATCAGTGGGATGAGGGAGACTGTTCACCTGCTGTGTACTCCTGTTTGGAAGTATTTAGATCCAGATTCTACTTAATGGGGTTTATATGGACTTTCTTCTCATAAATGGCCTGCCGTCTCCCTTCCTTTGAAGAGGATATGGGGATTCTGCTCTCTTTTCTTATTTACATGTAAATAATACATTGTTCTAAGTCTTTTTCATTAAAAATTTAAAACTTTTCCCATAAACTCTATACTTCTAAGGTGCCACCACCTTCTCTAGTAACTTACTGTGTAGTCTTGGGTCATTTCACTCACTTTTCTTTTTTTCTTTTCTTTTTCTTTTTTTTTTTTGAGACAGTCTCCCTCTGTTGCCCAGGCAGGAGCGCAGTGGCGTGATCTCCTCTCACCGCAAGCTCCACTTCCTGGGTTCATGCCATTCTCCTACCTCAGCCTCCCACGTAGCTGGGAGTACAGGCGGCCGCCACCATGCCAGGCTAATTTTTTTGTATTTTCAGTAGAGACGGGGTTTCACCGTGTTAGCCAAGATGGTCTCGATCTCCTGACCTCGTGATCCACCCACCTCGGCCTCCCAAAGTGCTGGGATTACAGGCGTGAGCCACTGCACCCGGCCCATTTCACTCACTTTTCTAATCTTTTTTTTTGGAAAGAGTTTCATCGTTGATGAGTTTTGCTCTGCTGCCCAGGCTGAAGAGCAGTGGCACCATCACAACTCATTGCAGCCACAACCTCCTGGGCTCAAGCGATCCTCTCACCTCACCCTCCTGAGTAGCTGGGACTACAGGTGCATGCCAGCACATCTGGCTAATATTTAAATTTTTTGTAGAGACAGGGTCTCACTTTGTGGCCTAGGCTGGTCTGTCACTCATGGGTTTAAGTGATTCTCCCATCTTGGCCTTACAAAGTGCTGGGATAACAGGTGTGAGCCACCATGCCTGGCCTCATTTTTCTAATTTTTTTTTTTTGTTGTTTTTTTGAGAGCCCAGGATCTCCCTGTCGCCCAGGCAGGAGTATAGTGGCAGGATCATGACTTACCACAATGTTGACAAACTAAAACAGATAATTATCTGTTTAAATATTTAAATTATTAAAGTATTTAAGTTATCTGTTGAAA |
| LV-cSERPINE2(Human) | GTCGTCCTTGGTGGAAGGAACCATGAACTGGCATCTCCCCCTCTTCCTCTTGGCCTCTGTGACGCTGCCTTCCATCTGCTCCCACTTCAATCCTCTGTCTCTCGAGGAACTAGGCTCCAACACGGGGATCCAGGTTTTCAATCAGATTGTGAAGTCGAGGCCTCATGACAACATCGTGATCTCTCCCCATGGGATTGCGTCGGTCCTGGGGATGCTTCAGCTGGGGGCGGACGGCAGGACCAAGAAGCAGCTCGCCATGGTGATGAGATACGGCGTAAATGGAGTTGGTAAAATATTAAAGAAGATCAACAAGGCCATCGTCTCCAAGAAGAATAAAGACATTGTGACAGTGGCTAACGCCGTGTTTGTTAAGAATGCCTCTGAAATTGAAGTGCCTTTTGTTACAAGGAACAAAGATGTGTTCCAGTGTGAGGTCCGGAATGTGAACTTTGAGGATCCAGCCTCTGCCTGTGATTCCATCAATGCATGGGTTAAAAATGAAACCAGGG |
| LV-cSERPINE2(Mouse) | GCTGTCCTTGTTGGAAGGAACCATGAATTGGCATTTTCCTTTCTTCATCTTGACCACAGTGACTTTATACTCTGTGCACTCCCAGTTCAACTCTCTGTCACTGGAGGAACTAGGCTCCAACACAGGGATCCAGGTCTTCAATCAGATCATCAAGTCACGGCCTCATGAGAACGTTGTTGTCTCCCCACATGGGATCGCGTCCATCTTGGGCATGCTGCAGCTCGGGGCTGACGGCAAGACAAAGAAGCAGCTCTCCACGGTGATGCGATATAATGTAAACGGAGTTGGTAAAGTGCTGAAGAAGATCAACAAGGCTATTGTCTCCAAGAAAAATAAAGACATTGTGACCGTGGCCAATGCTGTGTTTCTCAGGAATGGCTTTAAAATGGAAGTGCCTTTTGCAGTAAGGAACAAAGATGTGTTTCAGTGTGAAGTGCAGAATGTGAACTTCCAGGACCCAGCCTCTGCCTCTGAGTCCATCAATTTTTGGGTCAAAAATGAGACCAGGG |

**Supplementary Table S11.**

Effective sequences (5’-3’) of lentivirus plasmids-knockdown.

| Plasmids | 5’-3’ |
| --- | --- |
| si-NC | UUCUCCGAACGUGUCACGUTT |
| siDHX9-1 | GCCUCCAAGAAAGUCCAAUUUAUUGGACUUUCUUGGAGGCTT |
| siDHX9-2 | GAAGGAUUACUACUCAAGAAACUCGAGUUUCUUGAGUAGUAAUCCUUCUUUUTT |
| siQKI-1 | AAUUAGCAGAGUACGGAAATT |
| siQKI-2 | GAUUGUGACCGCAGACCGATT |
| siEIF4A3-1 | AGUGGAAUUCGAGACCAGCTT |
| siEIF4A3-2 | CAAUCAAGCAGAUCAUCAATT |
| siFUS-1 | AUGAAUGCAACCAGUGUAATT |
| siFUS-2 | CCAAUUCCUGAUCACCCAATT |
| siADAR1-1 | GGAUGCAAAUCAAGAGAAATT |
| siADAR1-2 | GGUGUUCACCCUACAAGAATT |
| siHnRNPM-1 | GAUUGACGUUCGAAUUGAUTT |
| siHnRNPM-2 | CGAUUUGGAUCUGGGAUGATT |
| siSTAT3-1 | GGAGCUGUUUAGAAACUUATT |
| siSTAT3-2 | GGUACAACAUGCUGACCAATT |
| sh-NC | CTTTCTCCGAACGTGTCACTT |
| shMALT-1(human) | GGGAGTATATGGGTTATTATT |
| shMALT-2(human) | GCAAATCTGTGTTGAACCATT |
| shMALT-1(Mouse) | GCAGCTACTTAGTATCAAATT |
| shMALT-2(Mouse) | GCAGCATGTTGTTGTTACATT |
| shcSERPINE2-1 (Human) | TCACAATCTGATTGAAAACCTTT |
| shcSERPINE2-2 (Human) | TCCATTTACGCCGTATCTCATTT |
| shcSERPINE2-1 (Mouse) | CAAGGACAGCCCCTGGTCTTT |
| shcSERPINE2-2 (Mouse) | GACAGCCCCTGGTCTCATTTT |

**Supplementary Table S12.**

miRNA mimics and inhibitor sequences (5’-3’).

| Name | | 5’-3’ |
| --- | --- | --- |
| miR-inhibitor-NC | Sense | CAGUACUUUUGUGUAGUACAA |
|  | Antisense | None |
| miR-513a-5p inhibitor | Sense | AUGACACCUCCCUGUGAA |
|  | Antisense | None |
| miR-mimic-NC | Sense | UUGUACUACACAAAAGUACUG |
|  | Antisense | CAGUACUUUUGUGUAGUACAA |
| miR-513a-5p mimic | Sense | UUCACAGGGAGGUGUCAU |
|  | Antisense | GACACCUCCCUGUGAAUU |
