# Supplementary figures and images for: Targeting tumor exosomal circular RNA cSERPINE2 suppresses breast cancer progression by modulating MALT1-NF-𝜅B-IL-6 axis of tumor-associated macrophages
Source: J Exp Clin Cancer Res. 2023 Feb 17;42:48. doi: 10.1186/s13046-023-02620-5 (PMC9936722; doi:10.1186/s13046-023-02620-5)

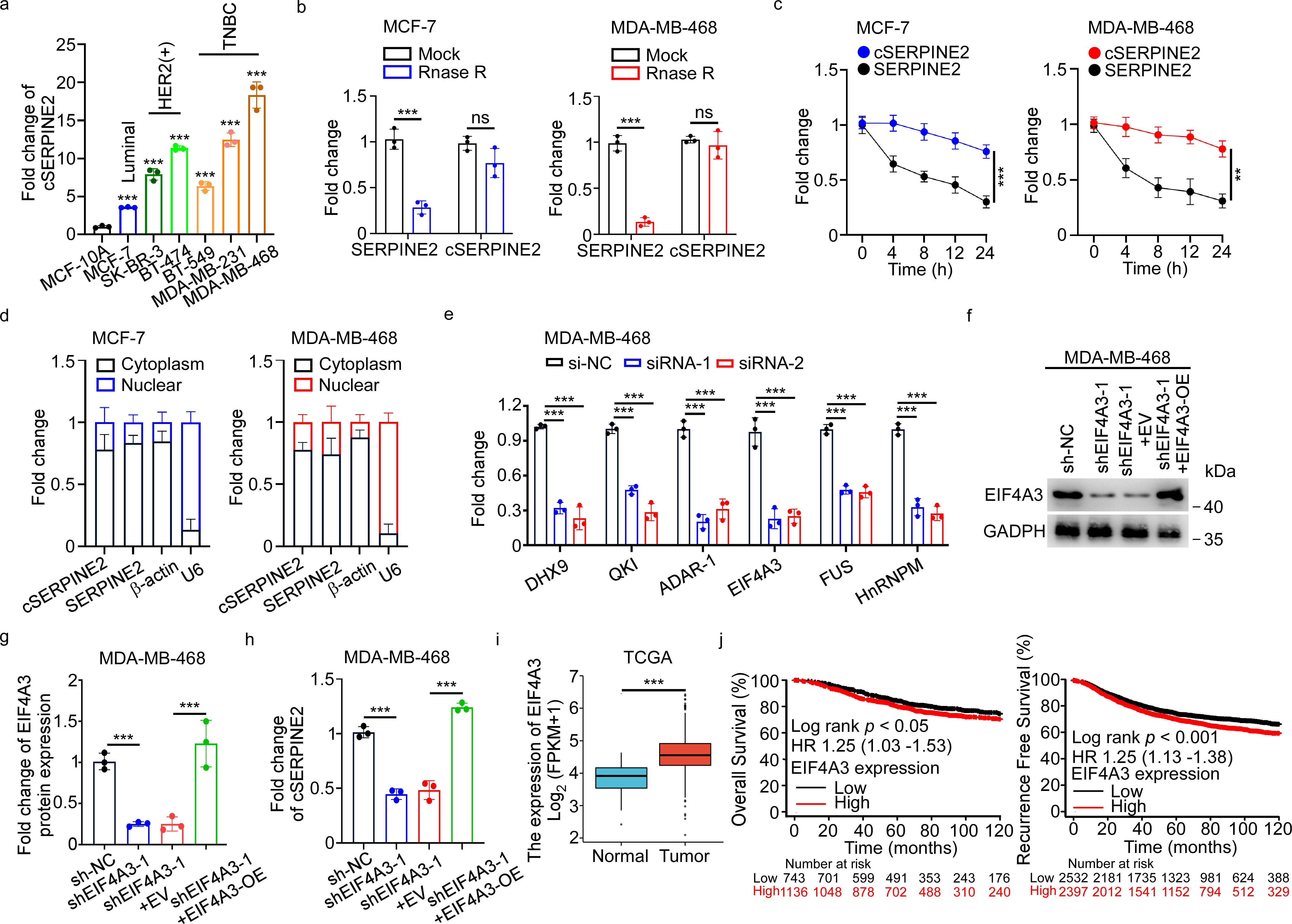

Supplement: Supplementary file 1 — Additional file 1: Fig. S1. Analysis of the expression and character of cSERPINE2 in breast cancercells. Fig. S2. cSERPINE2 reshaped the immune microenvironment of breast cancer. Fig. S3 Tumor exosomal cSERPINE2 targeted macrophages to promote theproliferation and invasion of breast cancer cells. Fig. S4. Tumor exosomal cSERPINE2 upregulated the MALT1 expressionin macrophages by sponging miR‑513a-5p. Fig. S5. Tumor exosomal cSERPINE2 promoted the secretion of IL-6 in TAMs via activating the NF-κB pathway to promote the progression of breast cancer. Fig. S6. IL-6 secreted by TAMs promoted the expression of CCL2 in breastcancer cells via activating the JAK2-STAT3 pathway. Fig. S7. Characteristics of si-cSERPINE2-NPs and therapeutic efficacy of si-cSERPINE2-NPs in breast cancer invitro. Fig. S8. Toxicity evaluation of systemic injection of si-cSERPINE2-NPs in breast cancer. Supplementary Table S1. Correlation between cSERPINE2 expression and clinicopathological features in 136 patients withbreast cancer. Supplementary Table S2. Univariate and multivariate analyses of OS in breast cancer (n=136). Supplementary Table S3. Univariate and multivariate analyses of RFS in breast cancer (n=136). Supplementary Table S4. The primers (5’-3’) used for RT-qPCR in this study. Supplementary Table S5. The probe sequences (5’-3’) for FISH. Supplementary Table S6. Primers for EIF4A3 RIP. Supplementary Table S7. Primers for CHIP. Supplementary Table S8. The probe sequences (5’-3’) for RNA pull-down assay. Supplementary Table S9. Primers for in vitro transcription. Supplementary Table S10. Effective sequences (5’-3’) of lentivirus plasmids-overexpression. Supplementary Table S11. Effective sequences (5’-3’) of lentivirus plasmids-knockdown. Supplementary Table S12. miRNA mimics and inhibitor sequences (5’-3’). [file 13046_2023_2620_MOESM1_ESM.zip › 13046_2023_2620_MOESM1_ESM/Supplementary Figure 1.jpg]

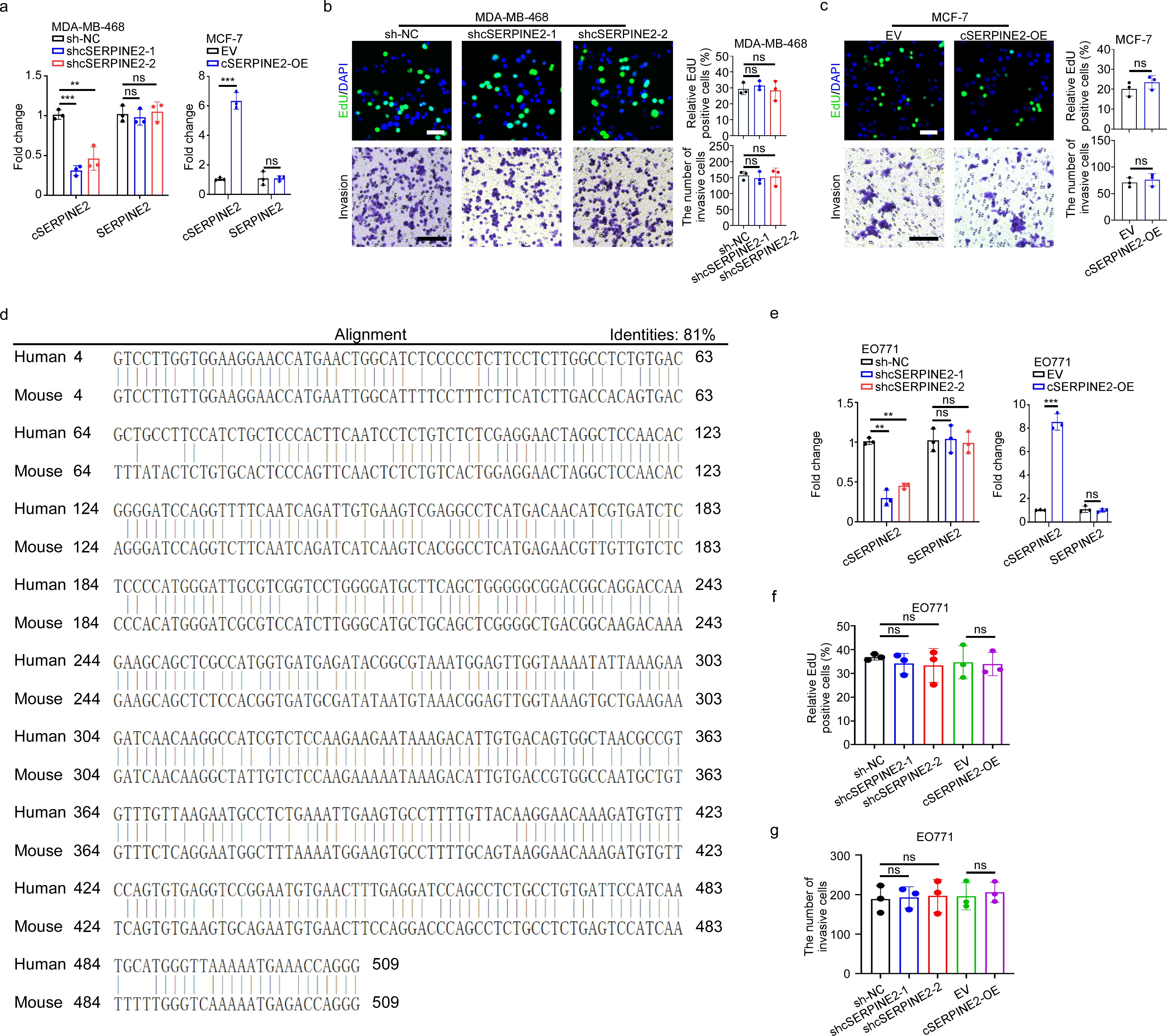

Supplement: Supplementary file 1 — Additional file 1: Fig. S1. Analysis of the expression and character of cSERPINE2 in breast cancercells. Fig. S2. cSERPINE2 reshaped the immune microenvironment of breast cancer. Fig. S3 Tumor exosomal cSERPINE2 targeted macrophages to promote theproliferation and invasion of breast cancer cells. Fig. S4. Tumor exosomal cSERPINE2 upregulated the MALT1 expressionin macrophages by sponging miR‑513a-5p. Fig. S5. Tumor exosomal cSERPINE2 promoted the secretion of IL-6 in TAMs via activating the NF-κB pathway to promote the progression of breast cancer. Fig. S6. IL-6 secreted by TAMs promoted the expression of CCL2 in breastcancer cells via activating the JAK2-STAT3 pathway. Fig. S7. Characteristics of si-cSERPINE2-NPs and therapeutic efficacy of si-cSERPINE2-NPs in breast cancer invitro. Fig. S8. Toxicity evaluation of systemic injection of si-cSERPINE2-NPs in breast cancer. Supplementary Table S1. Correlation between cSERPINE2 expression and clinicopathological features in 136 patients withbreast cancer. Supplementary Table S2. Univariate and multivariate analyses of OS in breast cancer (n=136). Supplementary Table S3. Univariate and multivariate analyses of RFS in breast cancer (n=136). Supplementary Table S4. The primers (5’-3’) used for RT-qPCR in this study. Supplementary Table S5. The probe sequences (5’-3’) for FISH. Supplementary Table S6. Primers for EIF4A3 RIP. Supplementary Table S7. Primers for CHIP. Supplementary Table S8. The probe sequences (5’-3’) for RNA pull-down assay. Supplementary Table S9. Primers for in vitro transcription. Supplementary Table S10. Effective sequences (5’-3’) of lentivirus plasmids-overexpression. Supplementary Table S11. Effective sequences (5’-3’) of lentivirus plasmids-knockdown. Supplementary Table S12. miRNA mimics and inhibitor sequences (5’-3’). [file 13046_2023_2620_MOESM1_ESM.zip › 13046_2023_2620_MOESM1_ESM/Supplementary Figure 2.jpg]

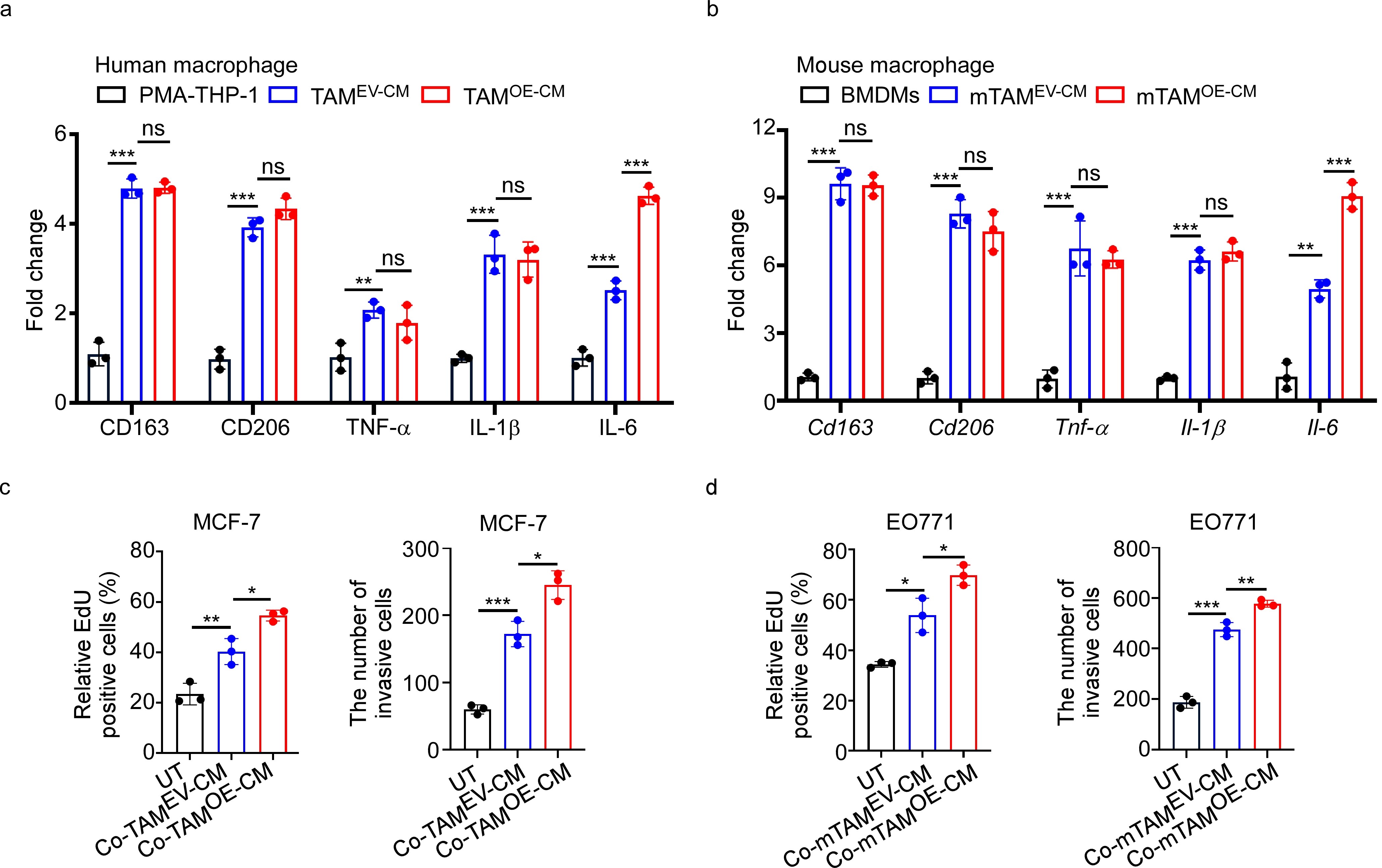

Supplement: Supplementary file 1 — Additional file 1: Fig. S1. Analysis of the expression and character of cSERPINE2 in breast cancercells. Fig. S2. cSERPINE2 reshaped the immune microenvironment of breast cancer. Fig. S3 Tumor exosomal cSERPINE2 targeted macrophages to promote theproliferation and invasion of breast cancer cells. Fig. S4. Tumor exosomal cSERPINE2 upregulated the MALT1 expressionin macrophages by sponging miR‑513a-5p. Fig. S5. Tumor exosomal cSERPINE2 promoted the secretion of IL-6 in TAMs via activating the NF-κB pathway to promote the progression of breast cancer. Fig. S6. IL-6 secreted by TAMs promoted the expression of CCL2 in breastcancer cells via activating the JAK2-STAT3 pathway. Fig. S7. Characteristics of si-cSERPINE2-NPs and therapeutic efficacy of si-cSERPINE2-NPs in breast cancer invitro. Fig. S8. Toxicity evaluation of systemic injection of si-cSERPINE2-NPs in breast cancer. Supplementary Table S1. Correlation between cSERPINE2 expression and clinicopathological features in 136 patients withbreast cancer. Supplementary Table S2. Univariate and multivariate analyses of OS in breast cancer (n=136). Supplementary Table S3. Univariate and multivariate analyses of RFS in breast cancer (n=136). Supplementary Table S4. The primers (5’-3’) used for RT-qPCR in this study. Supplementary Table S5. The probe sequences (5’-3’) for FISH. Supplementary Table S6. Primers for EIF4A3 RIP. Supplementary Table S7. Primers for CHIP. Supplementary Table S8. The probe sequences (5’-3’) for RNA pull-down assay. Supplementary Table S9. Primers for in vitro transcription. Supplementary Table S10. Effective sequences (5’-3’) of lentivirus plasmids-overexpression. Supplementary Table S11. Effective sequences (5’-3’) of lentivirus plasmids-knockdown. Supplementary Table S12. miRNA mimics and inhibitor sequences (5’-3’). [file 13046_2023_2620_MOESM1_ESM.zip › 13046_2023_2620_MOESM1_ESM/Supplementary Figure 3.jpg]

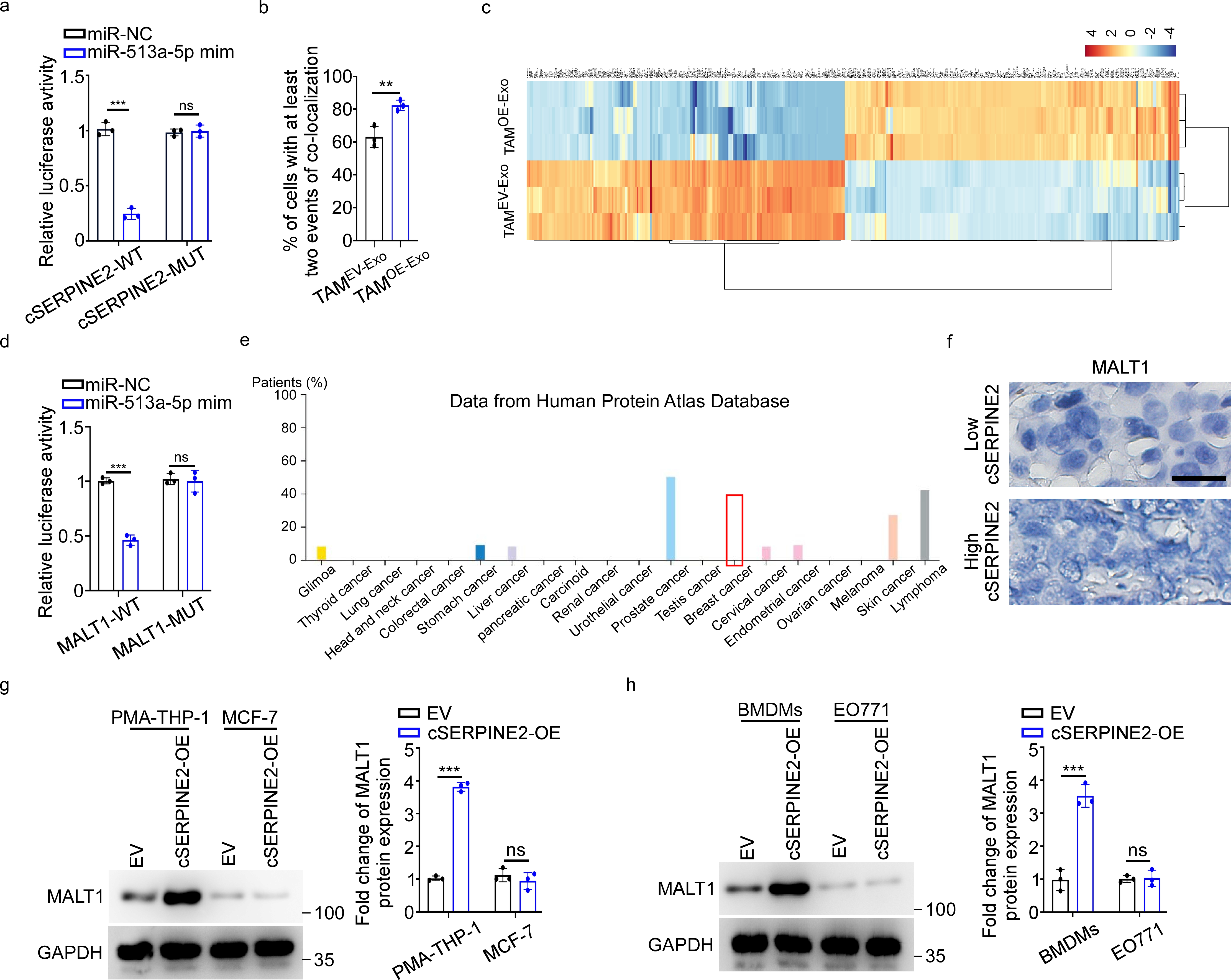

Supplement: Supplementary file 1 — Additional file 1: Fig. S1. Analysis of the expression and character of cSERPINE2 in breast cancercells. Fig. S2. cSERPINE2 reshaped the immune microenvironment of breast cancer. Fig. S3 Tumor exosomal cSERPINE2 targeted macrophages to promote theproliferation and invasion of breast cancer cells. Fig. S4. Tumor exosomal cSERPINE2 upregulated the MALT1 expressionin macrophages by sponging miR‑513a-5p. Fig. S5. Tumor exosomal cSERPINE2 promoted the secretion of IL-6 in TAMs via activating the NF-κB pathway to promote the progression of breast cancer. Fig. S6. IL-6 secreted by TAMs promoted the expression of CCL2 in breastcancer cells via activating the JAK2-STAT3 pathway. Fig. S7. Characteristics of si-cSERPINE2-NPs and therapeutic efficacy of si-cSERPINE2-NPs in breast cancer invitro. Fig. S8. Toxicity evaluation of systemic injection of si-cSERPINE2-NPs in breast cancer. Supplementary Table S1. Correlation between cSERPINE2 expression and clinicopathological features in 136 patients withbreast cancer. Supplementary Table S2. Univariate and multivariate analyses of OS in breast cancer (n=136). Supplementary Table S3. Univariate and multivariate analyses of RFS in breast cancer (n=136). Supplementary Table S4. The primers (5’-3’) used for RT-qPCR in this study. Supplementary Table S5. The probe sequences (5’-3’) for FISH. Supplementary Table S6. Primers for EIF4A3 RIP. Supplementary Table S7. Primers for CHIP. Supplementary Table S8. The probe sequences (5’-3’) for RNA pull-down assay. Supplementary Table S9. Primers for in vitro transcription. Supplementary Table S10. Effective sequences (5’-3’) of lentivirus plasmids-overexpression. Supplementary Table S11. Effective sequences (5’-3’) of lentivirus plasmids-knockdown. Supplementary Table S12. miRNA mimics and inhibitor sequences (5’-3’). [file 13046_2023_2620_MOESM1_ESM.zip › 13046_2023_2620_MOESM1_ESM/Supplementary Figure 4.jpg]

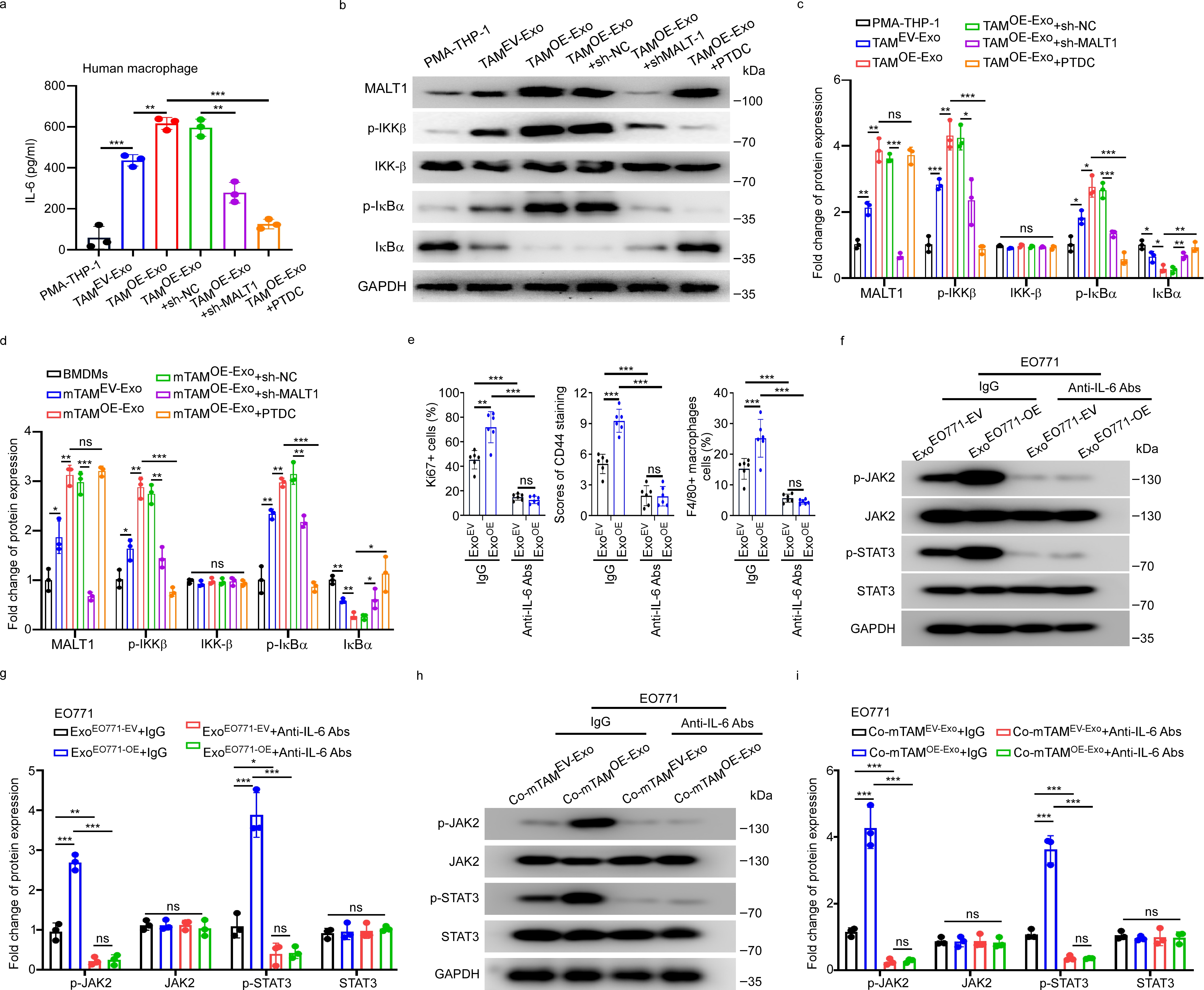

Supplement: Supplementary file 1 — Additional file 1: Fig. S1. Analysis of the expression and character of cSERPINE2 in breast cancercells. Fig. S2. cSERPINE2 reshaped the immune microenvironment of breast cancer. Fig. S3 Tumor exosomal cSERPINE2 targeted macrophages to promote theproliferation and invasion of breast cancer cells. Fig. S4. Tumor exosomal cSERPINE2 upregulated the MALT1 expressionin macrophages by sponging miR‑513a-5p. Fig. S5. Tumor exosomal cSERPINE2 promoted the secretion of IL-6 in TAMs via activating the NF-κB pathway to promote the progression of breast cancer. Fig. S6. IL-6 secreted by TAMs promoted the expression of CCL2 in breastcancer cells via activating the JAK2-STAT3 pathway. Fig. S7. Characteristics of si-cSERPINE2-NPs and therapeutic efficacy of si-cSERPINE2-NPs in breast cancer invitro. Fig. S8. Toxicity evaluation of systemic injection of si-cSERPINE2-NPs in breast cancer. Supplementary Table S1. Correlation between cSERPINE2 expression and clinicopathological features in 136 patients withbreast cancer. Supplementary Table S2. Univariate and multivariate analyses of OS in breast cancer (n=136). Supplementary Table S3. Univariate and multivariate analyses of RFS in breast cancer (n=136). Supplementary Table S4. The primers (5’-3’) used for RT-qPCR in this study. Supplementary Table S5. The probe sequences (5’-3’) for FISH. Supplementary Table S6. Primers for EIF4A3 RIP. Supplementary Table S7. Primers for CHIP. Supplementary Table S8. The probe sequences (5’-3’) for RNA pull-down assay. Supplementary Table S9. Primers for in vitro transcription. Supplementary Table S10. Effective sequences (5’-3’) of lentivirus plasmids-overexpression. Supplementary Table S11. Effective sequences (5’-3’) of lentivirus plasmids-knockdown. Supplementary Table S12. miRNA mimics and inhibitor sequences (5’-3’). [file 13046_2023_2620_MOESM1_ESM.zip › 13046_2023_2620_MOESM1_ESM/Supplementary Figure 5.jpg]

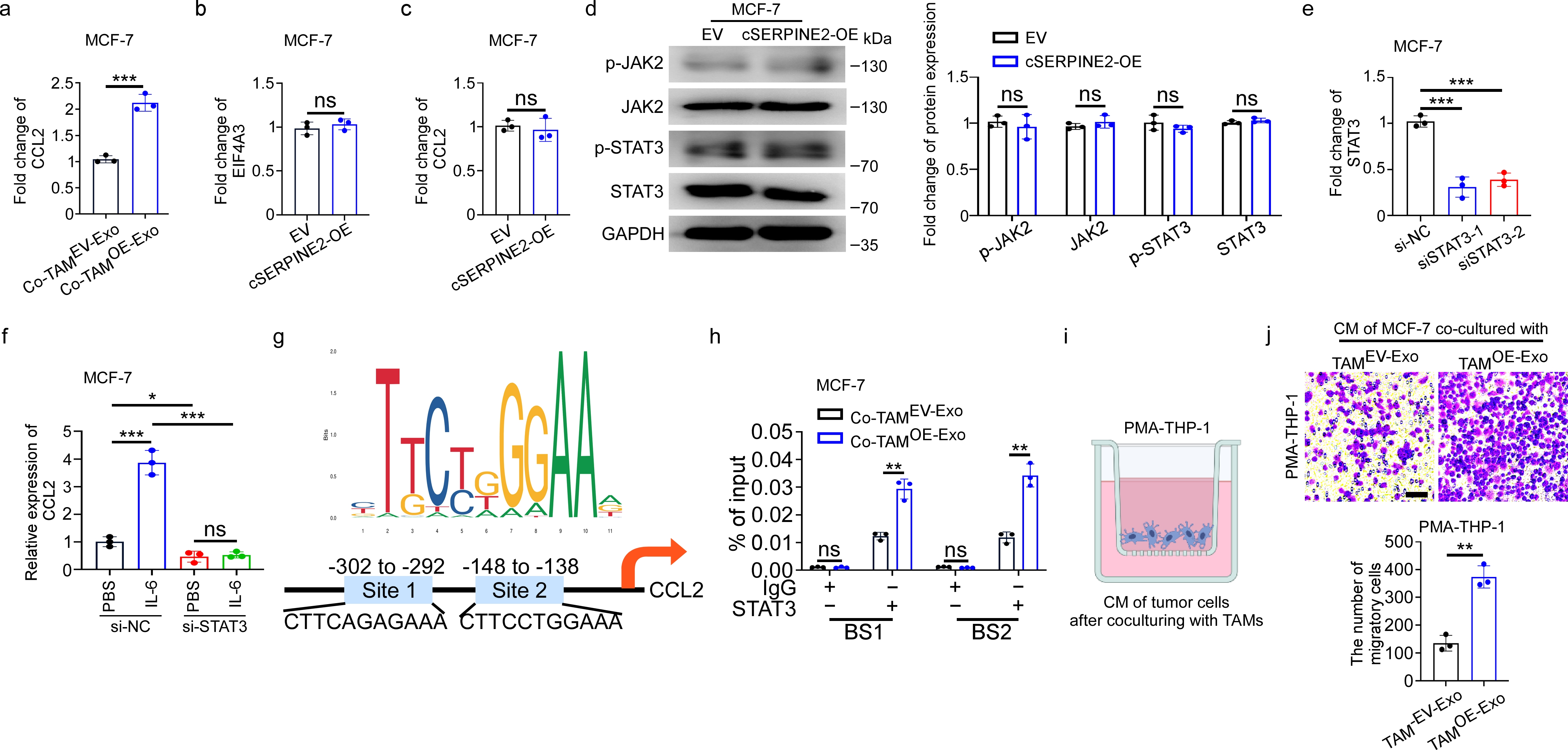

Supplement: Supplementary file 1 — Additional file 1: Fig. S1. Analysis of the expression and character of cSERPINE2 in breast cancercells. Fig. S2. cSERPINE2 reshaped the immune microenvironment of breast cancer. Fig. S3 Tumor exosomal cSERPINE2 targeted macrophages to promote theproliferation and invasion of breast cancer cells. Fig. S4. Tumor exosomal cSERPINE2 upregulated the MALT1 expressionin macrophages by sponging miR‑513a-5p. Fig. S5. Tumor exosomal cSERPINE2 promoted the secretion of IL-6 in TAMs via activating the NF-κB pathway to promote the progression of breast cancer. Fig. S6. IL-6 secreted by TAMs promoted the expression of CCL2 in breastcancer cells via activating the JAK2-STAT3 pathway. Fig. S7. Characteristics of si-cSERPINE2-NPs and therapeutic efficacy of si-cSERPINE2-NPs in breast cancer invitro. Fig. S8. Toxicity evaluation of systemic injection of si-cSERPINE2-NPs in breast cancer. Supplementary Table S1. Correlation between cSERPINE2 expression and clinicopathological features in 136 patients withbreast cancer. Supplementary Table S2. Univariate and multivariate analyses of OS in breast cancer (n=136). Supplementary Table S3. Univariate and multivariate analyses of RFS in breast cancer (n=136). Supplementary Table S4. The primers (5’-3’) used for RT-qPCR in this study. Supplementary Table S5. The probe sequences (5’-3’) for FISH. Supplementary Table S6. Primers for EIF4A3 RIP. Supplementary Table S7. Primers for CHIP. Supplementary Table S8. The probe sequences (5’-3’) for RNA pull-down assay. Supplementary Table S9. Primers for in vitro transcription. Supplementary Table S10. Effective sequences (5’-3’) of lentivirus plasmids-overexpression. Supplementary Table S11. Effective sequences (5’-3’) of lentivirus plasmids-knockdown. Supplementary Table S12. miRNA mimics and inhibitor sequences (5’-3’). [file 13046_2023_2620_MOESM1_ESM.zip › 13046_2023_2620_MOESM1_ESM/Supplementary Figure 6.jpg]

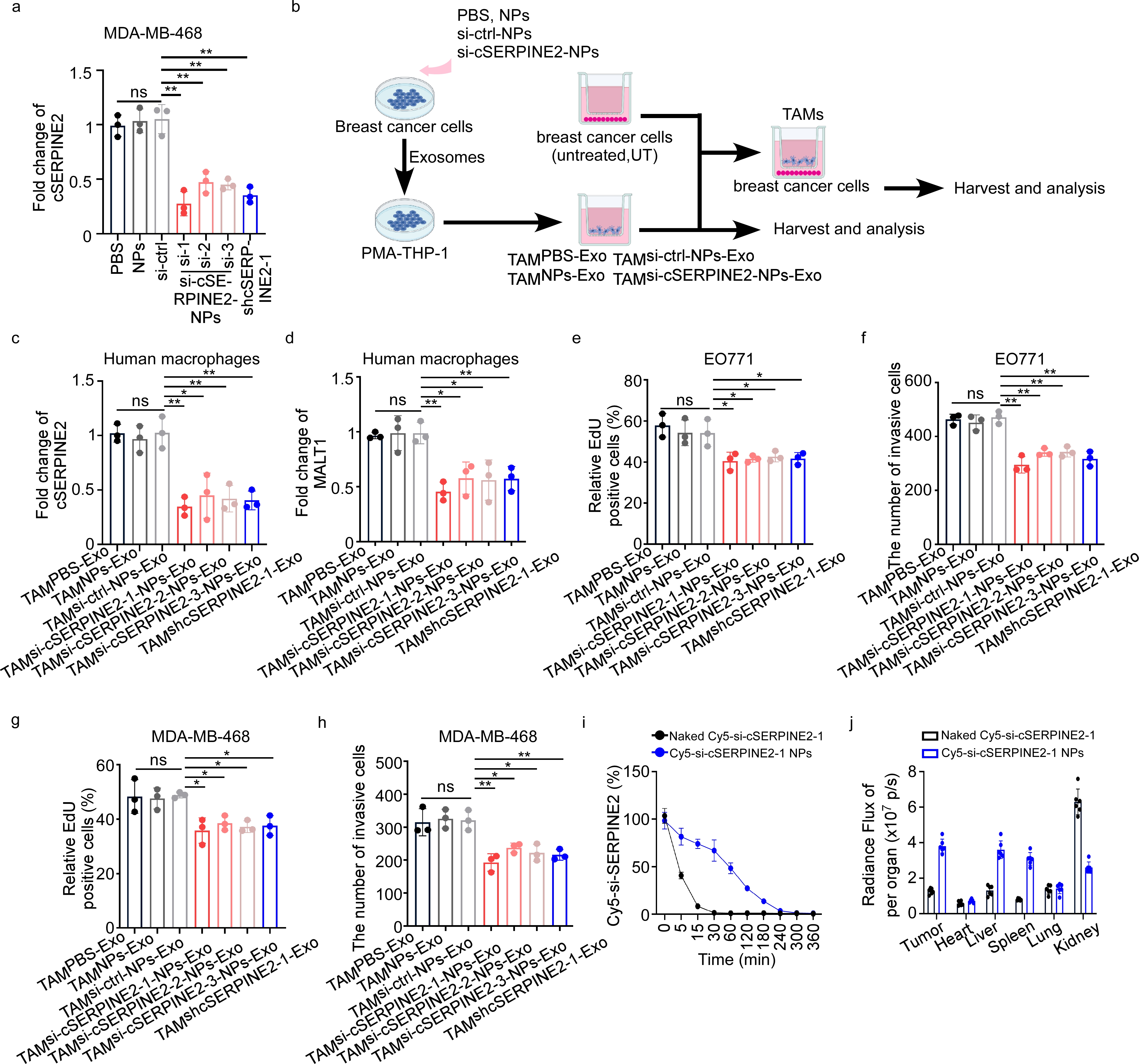

Supplement: Supplementary file 1 — Additional file 1: Fig. S1. Analysis of the expression and character of cSERPINE2 in breast cancercells. Fig. S2. cSERPINE2 reshaped the immune microenvironment of breast cancer. Fig. S3 Tumor exosomal cSERPINE2 targeted macrophages to promote theproliferation and invasion of breast cancer cells. Fig. S4. Tumor exosomal cSERPINE2 upregulated the MALT1 expressionin macrophages by sponging miR‑513a-5p. Fig. S5. Tumor exosomal cSERPINE2 promoted the secretion of IL-6 in TAMs via activating the NF-κB pathway to promote the progression of breast cancer. Fig. S6. IL-6 secreted by TAMs promoted the expression of CCL2 in breastcancer cells via activating the JAK2-STAT3 pathway. Fig. S7. Characteristics of si-cSERPINE2-NPs and therapeutic efficacy of si-cSERPINE2-NPs in breast cancer invitro. Fig. S8. Toxicity evaluation of systemic injection of si-cSERPINE2-NPs in breast cancer. Supplementary Table S1. Correlation between cSERPINE2 expression and clinicopathological features in 136 patients withbreast cancer. Supplementary Table S2. Univariate and multivariate analyses of OS in breast cancer (n=136). Supplementary Table S3. Univariate and multivariate analyses of RFS in breast cancer (n=136). Supplementary Table S4. The primers (5’-3’) used for RT-qPCR in this study. Supplementary Table S5. The probe sequences (5’-3’) for FISH. Supplementary Table S6. Primers for EIF4A3 RIP. Supplementary Table S7. Primers for CHIP. Supplementary Table S8. The probe sequences (5’-3’) for RNA pull-down assay. Supplementary Table S9. Primers for in vitro transcription. Supplementary Table S10. Effective sequences (5’-3’) of lentivirus plasmids-overexpression. Supplementary Table S11. Effective sequences (5’-3’) of lentivirus plasmids-knockdown. Supplementary Table S12. miRNA mimics and inhibitor sequences (5’-3’). [file 13046_2023_2620_MOESM1_ESM.zip › 13046_2023_2620_MOESM1_ESM/Supplementary Figure 7.jpg]

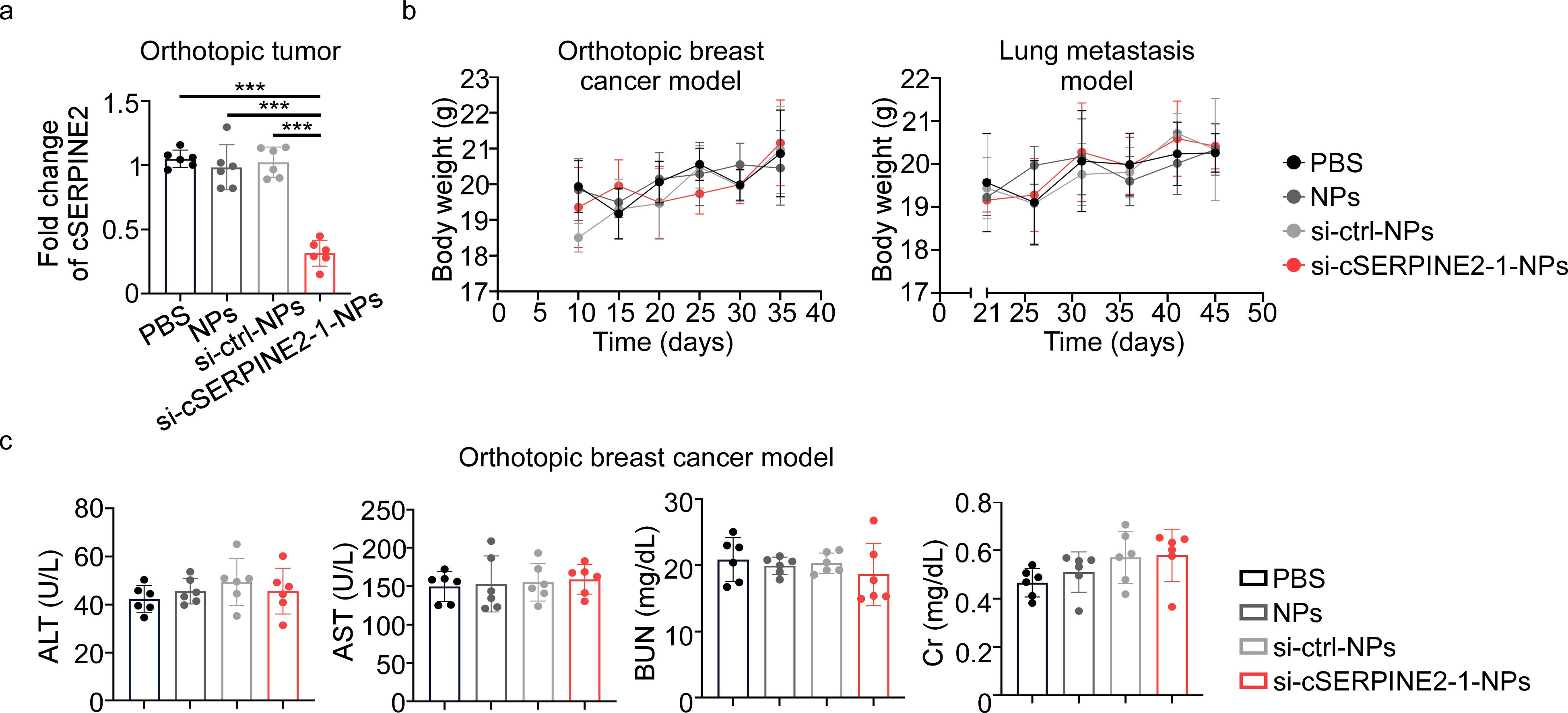

Supplement: Supplementary file 1 — Additional file 1: Fig. S1. Analysis of the expression and character of cSERPINE2 in breast cancercells. Fig. S2. cSERPINE2 reshaped the immune microenvironment of breast cancer. Fig. S3 Tumor exosomal cSERPINE2 targeted macrophages to promote theproliferation and invasion of breast cancer cells. Fig. S4. Tumor exosomal cSERPINE2 upregulated the MALT1 expressionin macrophages by sponging miR‑513a-5p. Fig. S5. Tumor exosomal cSERPINE2 promoted the secretion of IL-6 in TAMs via activating the NF-κB pathway to promote the progression of breast cancer. Fig. S6. IL-6 secreted by TAMs promoted the expression of CCL2 in breastcancer cells via activating the JAK2-STAT3 pathway. Fig. S7. Characteristics of si-cSERPINE2-NPs and therapeutic efficacy of si-cSERPINE2-NPs in breast cancer invitro. Fig. S8. Toxicity evaluation of systemic injection of si-cSERPINE2-NPs in breast cancer. Supplementary Table S1. Correlation between cSERPINE2 expression and clinicopathological features in 136 patients withbreast cancer. Supplementary Table S2. Univariate and multivariate analyses of OS in breast cancer (n=136). Supplementary Table S3. Univariate and multivariate analyses of RFS in breast cancer (n=136). Supplementary Table S4. The primers (5’-3’) used for RT-qPCR in this study. Supplementary Table S5. The probe sequences (5’-3’) for FISH. Supplementary Table S6. Primers for EIF4A3 RIP. Supplementary Table S7. Primers for CHIP. Supplementary Table S8. The probe sequences (5’-3’) for RNA pull-down assay. Supplementary Table S9. Primers for in vitro transcription. Supplementary Table S10. Effective sequences (5’-3’) of lentivirus plasmids-overexpression. Supplementary Table S11. Effective sequences (5’-3’) of lentivirus plasmids-knockdown. Supplementary Table S12. miRNA mimics and inhibitor sequences (5’-3’). [file 13046_2023_2620_MOESM1_ESM.zip › 13046_2023_2620_MOESM1_ESM/Supplementary Figure 8.jpg]
